# Facile synthesis of functionalized tetrahydroquinolines via domino Povarov reactions of arylamines, methyl propiolate and aromatic aldehydes

**DOI:** 10.3762/bjoc.8.211

**Published:** 2012-10-26

**Authors:** Jing Sun, Hong Gao, Qun Wu, Chao-Guo Yan

**Affiliations:** 1 College of Chemistry & Chemical Engineering, Yangzhou University, Yangzhou 225002, China

**Keywords:** *β*-enamino ester, domino reaction, electron-deficient alkyne, Povarov reaction, tetrahydroquinoline

## Abstract

In the presence of *p*-toluenesulfonic acid as catalyst the domino reaction of arylamines, methyl propiolates and aromatic aldehydes in ethanol proceeded smoothly to give polysubstituted 1,2,3,4-tetrahydroquinolines in moderate yields. The reaction is believed to involve the Povarov reaction of in situ generated *β*-enamino ester with the in situ formed aromatic imine.

## Introduction

The Diels–Alder reaction is recognized as a powerful reaction in synthetic strategies for the production of natural and unnatural polycarbocycles and polyheterocycles [1–3]. Therefore, the hetero-Diels–Alder reactions and domino reaction procedures have been developed as the most powerful synthetic routes to construct oxygen or nitrogen-containing six-membered heterocycles [4–8]. In recent years, the imine Diels–Alder reaction (also known as Povarov reaction) and the [4 + 2] cycloaddition reaction of imines (obtained from the corresponding aromatic aldehyde and aniline derivatives) with alkenes have become efficient strategies for the generation of *N*-heterocycles [9–11]. In these reactions the alkene must be electron-rich, which means that functional groups attached to the alkene should be able to donate electrons. Among the electron-rich dienophiles, vinyl enol ethers, vinyl enamides, vinyl sulfides, cyclopentadienes, indenes, alkynes and enamines have been mostly used in this method [12–22]. β-Enamino esters [23–26], which may be readily generated in situ by the addition of a primary amine to electron-deficient alkynes, such as methyl propiolate or dimethyl acetylenedicarboxylate [27–30], have shown versatile reactivity and have been widely used as key intermediates in a number of domino reactions to construct heterocyclic systems [31–42]. However, a survey of literature reveals that a Povarov reaction with in situ generated β*-*enamino ester as dienophile has not been reported until now. Recently, Zhu and Masson successfully developed three-component Povarov reactions using enamides as dienophiles leading to a highly efficient synthesis of enantiomerically enriched 4-amino-tetrahydroquinolines [43–44]. In this work our aim is to describe the domino Povarov reaction with both in situ formed aldimine and in situ generated β*-*enamino ester for the facile synthesis of the functionalized tetrahydroquinoline.

## Results and Discussion

**Scheme 1:**
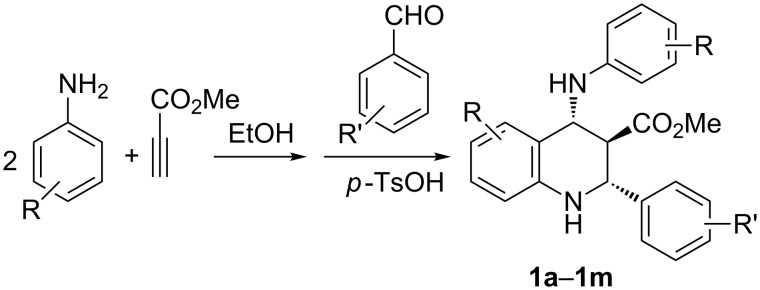
Synthesis of polysubstituted tetrahydroquinolines **1a**–**1m**.

It is known that the formation of β*-*enamino ester by the reaction of arylamine with methyl propiolate requires a relative long time [38–42]. Thus, under our previously established reaction conditions a molar excess of *p*-toludine (4.0 mmol) and methyl propiolate (2.0 mmol) reacted in ethanol at room temperature overnight to give the desired β*-*enamino ester. Then, benzaldehyde and *p*-toluenesulfonic acid was introduced in the reaction system and the sequential reaction was finished in 48 hours at room temperature monitored by TLC. After workup we were pleased to find that the functionalized tetrahydroquinoline **1c** was prepared in 63% yield (Scheme 1). The tetrahydroquinoline was obviously formed by the reaction of the previously formed β*-*enamino ester with in situ generated *N*-aryl aldimine. Thus, a domino Povarov reaction is successfully established. Similarly, various arylamines and aromatic aldehydes were used in the reaction under the same conditions. The results are summarized in Table 1. All the reactions proceeded smoothly to afford the corresponding functionalized tetrahydroquinolines (**1a**–**1m**) in moderate to good yields (41–67%). Arylamines and aromatic aldehydes with an electron-donating alkyl or methoxy group and with weak electron-withdrawing chloro or bromo groups reacted efficiently to give the expected products.

**Table 1:** Synthesis of the polysubstituted tetrahydroquinolines **1a**–**1m**.

Entry	Compound	R	R’	Yield (%)

1	**1a**	H	H	64
2	**1b**	H	*p-*Cl	48
3	**1c**	*p*-CH_3_	H	63
4	**1d**	*p*-CH_3_	*p*-CH_3_O	46
5	**1e**	*p*-CH_3_	*p*-CH(CH_3_)_2_	41
6	**1f**	*p*-CH_3_	*p-*Cl	48
7	**1g**	*p*-CH_3_O	*p*-CH_3_	51
8	**1h**	*p-*Cl	H	59
9	**1i**	*p-*Cl	*p*-CH_3_	66
10	**1j**	*p-*Cl	*p*-CH_3_O	54
11	**1k**	*p-*Cl	*p-*Cl	61
12	**1l**	*p*-Br	H	67
13	**1m**	*p*-Br	*p*-CH_3_	56

**Figure 1:**
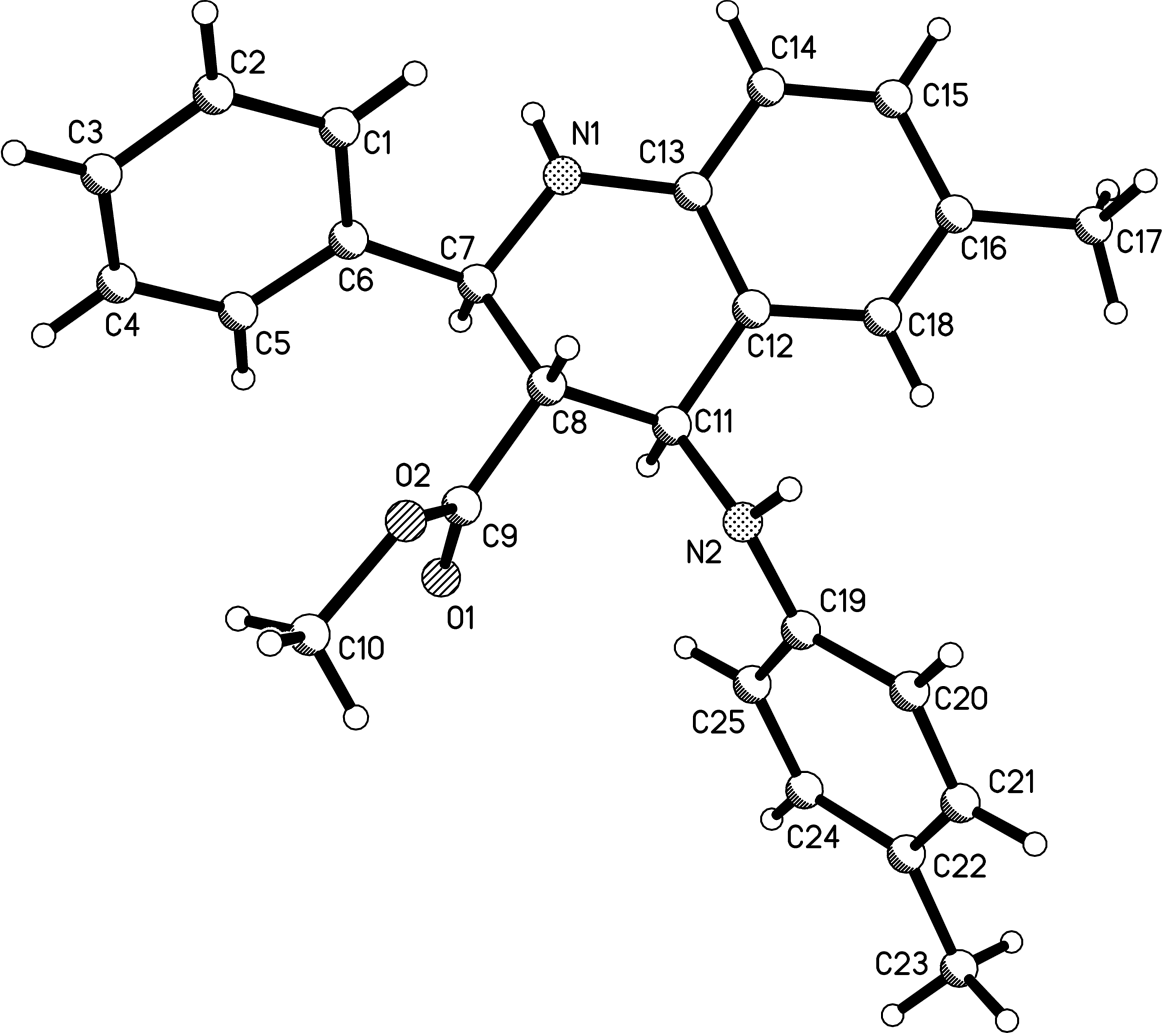
Molecular structure of compound **1c**.

The structures of the above-prepared functionalized tetrahydroquinolines **1a**–**1m** were characterized by ^1^H and ^13^C NMR, MS, HRMS and IR spectra and were further confirmed by single-crystal X-ray diffraction performed for the compound **1c** (Figure 1). The ^1^H NMR spectra of compounds **1a**–**1m** usually show two doublets and one triplet shift for the three cyclic CH units at the tetrahydroquinoline core. For example, in the ^1^H NMR spectrum of **1a** the triplet at 3.09 ppm belongs to the proton at the 3-position, while the doublets at 4.76 and 5.24 ppm are clearly the protons at the 2- and 4-position. The three groups at the 2-, 3- and 4-position of tetrahydroquinoline could be of either *cis* or *trans* configuration. Thus, several diastereoisomers would exist in tetrahydroquinolines **1a**–**1m**. ^1^H NMR spectra clearly indicates that there is only one diastereoisomer in the compounds **1a**–**1m**. Single crystal structure of compound **1c** (Figure 1) clearly showed that three groups at the 2-, 3- and 4-positions exist in *trans*-configuration. Thus, by analyzing ^1^H NMR spectra and the single crystal structure we could conclude that the prepared tetrahydroquinolines **1a**–**1m** exist in (2,3)-*trans*-(3,4)-*trans*-configuration, which also means that this domino Povarov reaction is a highly stereoselective reaction.

**Scheme 2:**
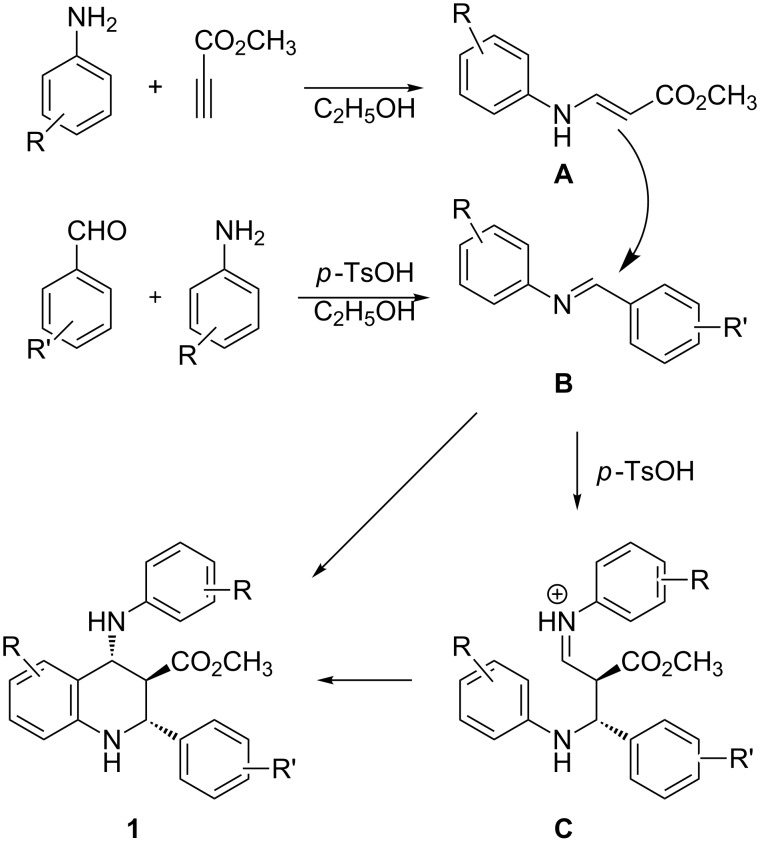
The proposed mechanism of domino Povarov reaction.

A reaction mechanism for this domino Povarov reaction is briefly outlined in Scheme 2 based on the published domino Povarov reaction [45–49]. At first, arylamine adds to methyl propiolate to form the β*-*enamino ester **A**. Secondly, excess arylamine reacts with the aromatic aldehyde to form the *N*-aryl aldimine **B** in the presence of *p*-toluenesulfonic acid as catalyst. Thirdly, the Mannich type addition of intermediate **A** with the acid-promoted *N*-aryl aldimine **B** gives the intermediate **C**. Lastly, the intramolecular electrophilic aromatic substitution at the ortho position of the activated *N*-aryl ring gives the final tetrahydroquinoline **1**. On the other hand, the concerted imine-Diels–Alder reaction of *N*-aryl aldimine **B** as the *N*-hetero diene with β*-*enamino ester **A** as the dienophile may give directly the tetrahydroquinoline **1**. At present it is very difficult to distinguish these two reaction paths. It may be due to the fact that the four sequential reactions are all retro equilibrium reactions; the thermodynamically stable *trans*-isomer is obtained as the final separated product.

In the reaction mechanism shown in Scheme 2, arylamine reacts not only with methyl propiolate to form a β-enamino ester, but it also reacts with an aromatic aldehyde to form an imine. We envisioned that two kinds of aryl amines could be used in the reaction to give more polysubstituted tetrahydroquinolines. Thus, after the addition reaction of one kind of arylamine with methyl propiolate had been completed, the aromatic aldehyde and the second arylamine were introduced to the reaction system. By using this method the functionalized tetrahydroquinolines **2a**–**2e** were successfully obtained in good yields (Table 2). This result showed that this domino Povarov reaction has a broad substrate scope.

**Table 2:** Synthesis of functionalized tetrahydroquinolines **2a**–**2e**.

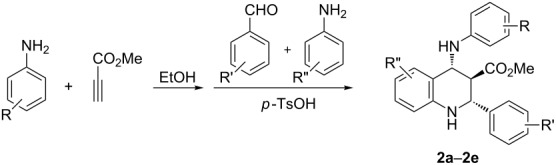					

Entry	Compound	R	R’	R’’	Yield (%)

1	**2a**	*p*-CH_3_	*p-*Cl	*p-*Cl	60
2	**2b**	*p*-CH_3_O	*p-*Cl	*p-*Cl	63
3	**2c**	*p*-CH_3_	*p-*Cl	H	56
4	**2d**	*p*-CH_3_O	*p*-Br	*p-*Cl	57
5	**2e**	*p*-CH_3_	*m-*CH_3_	*p-*Cl	55

## Conclusion

In summary we investigated the domino three-component reaction of aromatic aldehydes, arylamines and methyl propiolates, and successfully established the imino Diels–Alder reaction with β-enamino ester as dienophile. This reaction provides a convenient and stereoselective procedure for the preparation of 2-aryl-4-arylamino-1,2,3,4-tetrahydroquinoline-3-carboxylates in satisfactory yields. Furthermore, we successfully developed the domino Povarov reaction with in situ generated β*-*enamino ester as dienophiles. This methodology is potentially useful in the synthesis of tetrahydroquinoline derivatives.

## Experimental

**Reagents and apparatus.** All reactions were monitored by TLC. Melting points were taken on a hot-plate microscope apparatus. IR spectra were obtained on a Bruker Tensor 27 spectrometer (KBr disc). NMR spectra were recorded with a Bruker AV-600 spectrometer with CDCl_3_ as solvent and TMS as internal standard (600 and 150 MHz for ^1^H and ^13^C NMR spectra, respectively). HPLC/MS were measured on a Fennigan LCQ Deca XP MAX instrument. High-resolution mass (ESI) were obtained with a Bruker MicroTOF spectrometer. X-ray data were collected on a Bruker Smart APEX-2 CCD diffractometer. Aromatic aldehydes, arylamines, methyl propiolate and other reagents were commercial reagents and used as received. Solvents were purified by standard techniques.

**General procedure for the synthesis of polysubstituted tetrahydroquinolines.** A solution of arylamine (4.0 mmol) and methyl propiolate (2.0 mmol, 0.168 g) in 5 mL ethanol was stirred at room temperature overnight. Then, the aromatic aldehyde (2.0 mmol) and *p*-toluenelsulfonic acid (0.5 mmol) were added. The mixture was stirred at room temperature for an additional 48 h. The resulting precipitate was collected and washed with cold ethanol to give the solid product, which was subjected to thin-layer chromatography with light petroleum and ethyl acetate (v/v, 10:1) as developing reagent to give the pure product for analysis.

## Supporting Information

Experimental details and detailed spectroscopic data including crystallographic data (CIF) of all new compounds are available as Supporting Information. Single crystal data for compound **1c** (CCDC 890916) have been deposited in the Cambridge Crystallographic Data Centre, 12 Union Road, Cambridge, CB2 1EZ, UK (Fax: +44-1223-336033; e-mail: deposit@ccdc.cam.ac.uk or www: http://www.ccdc.cam.ac.uk).

File 1 General experimental methods and characterization of compounds.

File 2 X-ray crystallographic data of **1c**.
