# Supplementary material for: Facile synthesis of functionalized tetrahydroquinolines via domino Povarov reactions of arylamines, methyl propiolate and aromatic aldehydes
Source: Beilstein J Org Chem. 2012 Oct 26;8:1839–43. doi: 10.3762/bjoc.8.211 (PMC3511020; doi:10.3762/bjoc.8.211)

Supporting Information

for

Facile synthesis of functionalized tetrahydroquinolines via domino Povarov reactions of arylamines, methyl propiolate and aromatic aldehydes

Jing Sun, Hong Gao, Qun Wu and Chao-Guo Yan*

Address: College of Chemistry & Chemical Engineering, Yangzhou University, Yangzhou 225002, China

Email: Chao-Guo Yan* - cgyan@yzu.edu.cn

* Corresponding author

General experimental methods and characterization of compounds

Methyl 2-phenyl-4-phenylamino-1,2,3,4-tetrahydroquinoline-3-carboxylate (1a): White solid, 64%, mp 146–148℃; ^{1}H NMR (600 MHz, $CDCl_3$) δ: 7.39 (brs, 2H, ArH), 7.33–7.32 (m, 4H, ArH), 7.15 (brs, 2H, ArH), 7.08 (t, *J* = 7.2 Hz, 1H, ArH), 6.71–6.66 (m, 4H, ArH), 6.57 (d, *J* = 7.8 Hz, 1H, ArH), 5.24 (d, *J* = 9.6 Hz, 1H, CH), 4.76 (d, *J* = 9.6 Hz, 1H, CH), 3.31 (s, 3H, OCH_3), 3.09 (t, *J* = 9.6 Hz, 1H, CH); ^{13}C NMR (150 MHz, $CDCl_3$) δ: 172.9, 147.6, 143.8, 140.3, 129.4, 128.8, 128.5, 127.7, 127.2, 122.9, 118.1, 118.0, 114.1, 113.5, 59.0, 53.9, 53.6, 51.8; IR(KBr) υ: 3389, 2850, 1717, 1599, 1526, 1487, 1443, 1360, 1325, 1255, 1170, 750cm^{-1}; MS (*m/z*): 357.20 ($[M+1]^+$) 100%; HRMS (ESI) Calcd for $C_{23}H_{22}N_2NaO_2$ ($[M+Na]^+$): 381.1578. Found: 381.1553.

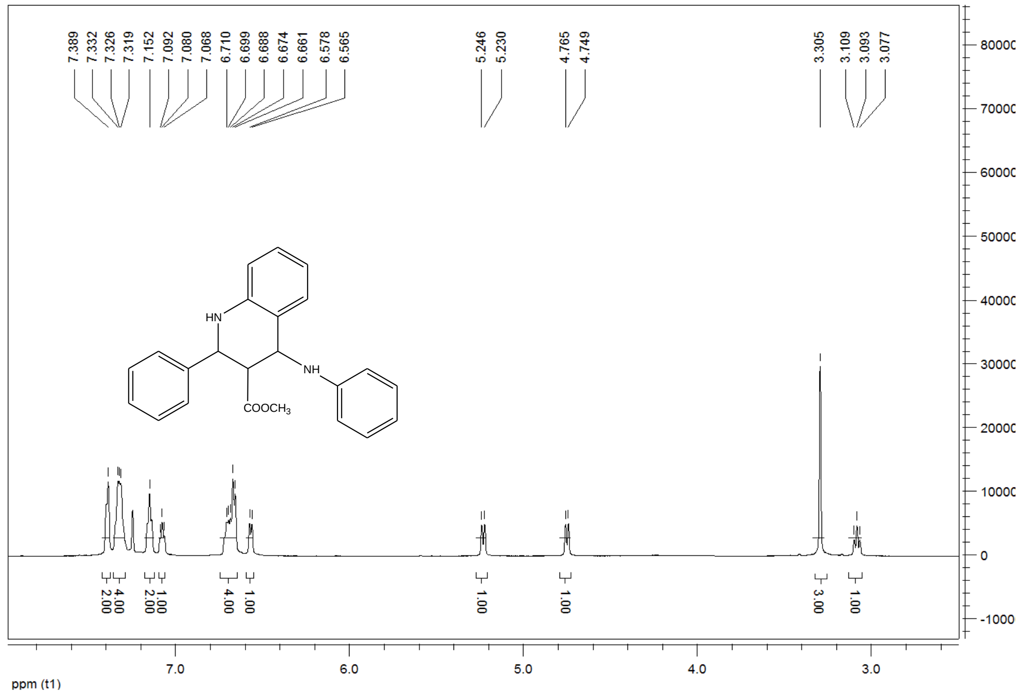

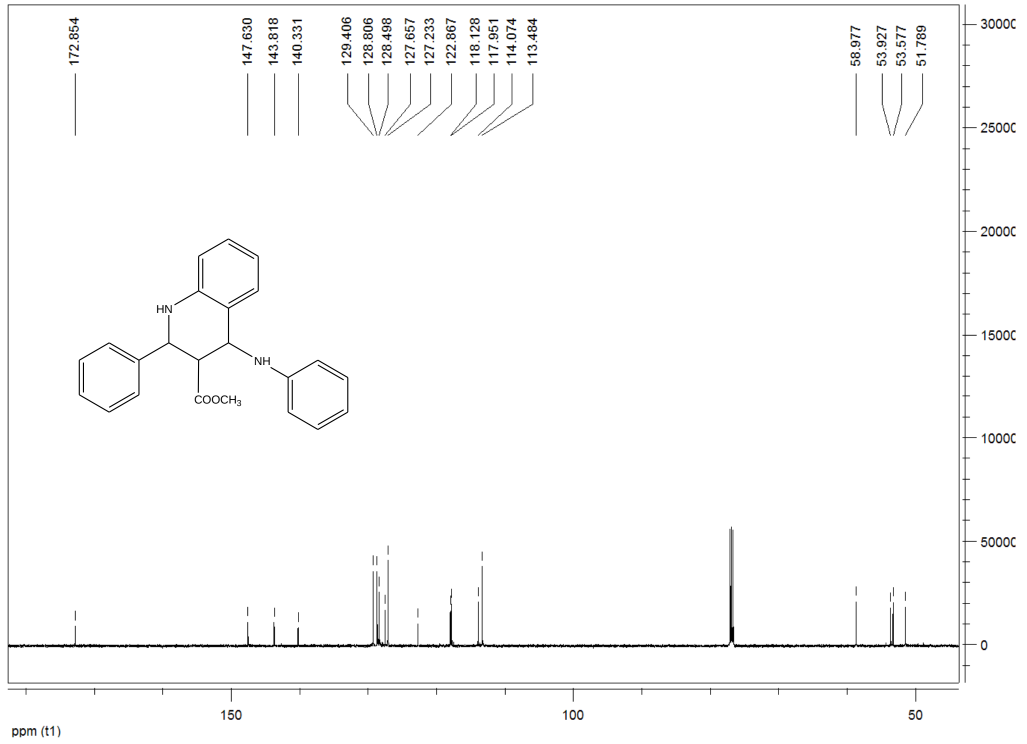

Methyl 2-(4-chlorophenyl)-4-phenylamino-1,2,3,4-tetrahydroquinoline-3-carboxylate (1b):

White solid, 48%, mp 175–177℃; ^{1}H NMR (600 MHz, $CDCl_3$) δ: 7.35–7.30 (m, 5H, ArH), 7.16 (t, *J* = 7.8 Hz, 2H, ArH), 7.09 (t, *J* = 7.8 Hz, 1H, ArH), 6.73–6.66 (m, 4H, ArH), 6.58 (d, *J* = 7.8 Hz, 1H, ArH), 5.23 (d, *J* = 10.2 Hz, 1H, CH), 4.74 (d, *J* = 10.2 Hz, 1H, CH), 3.32 (s, 3H, OCH_3), 3.04 (t, *J* = 10.2 Hz, 1H, CH); ^{13}C NMR (150 MHz, $CDCl_3$) δ: 172.7, 147.5, 143.6, 138.9, 134.2, 129.4, 129.0, 128.7, 128.6, 127.6, 122.9, 118.4, 118.1, 114.2, 113.5, 58.4, 54.0, 53.6, 51.9; IR(KBr) υ: 3397, 3027, 2948, 1724, 1601, 1524, 1485, 1439, 1367, 1304, 1256, 1174, 1090, 1006, 828, 752cm^{-1}; MS (*m/z*): 391.00 ($[M+1]^+$) 100%; HRMS (ESI) Calcd for $C_{23}H_{21}ClN_2NaO_2$ ($[M+Na]^+$): 415.1189. Found: 415.1188.

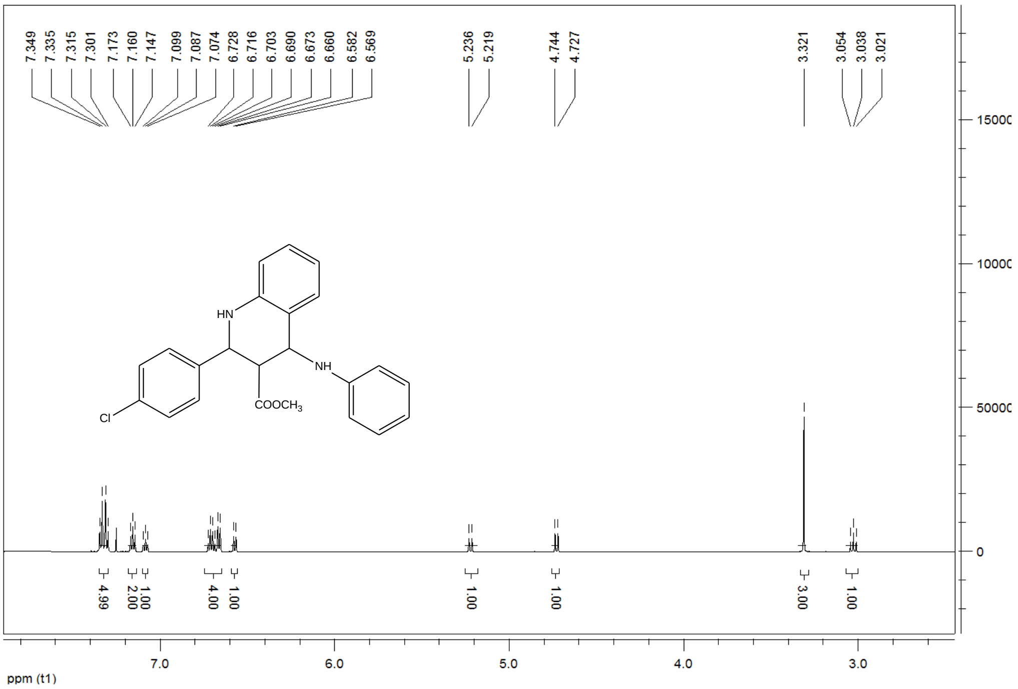

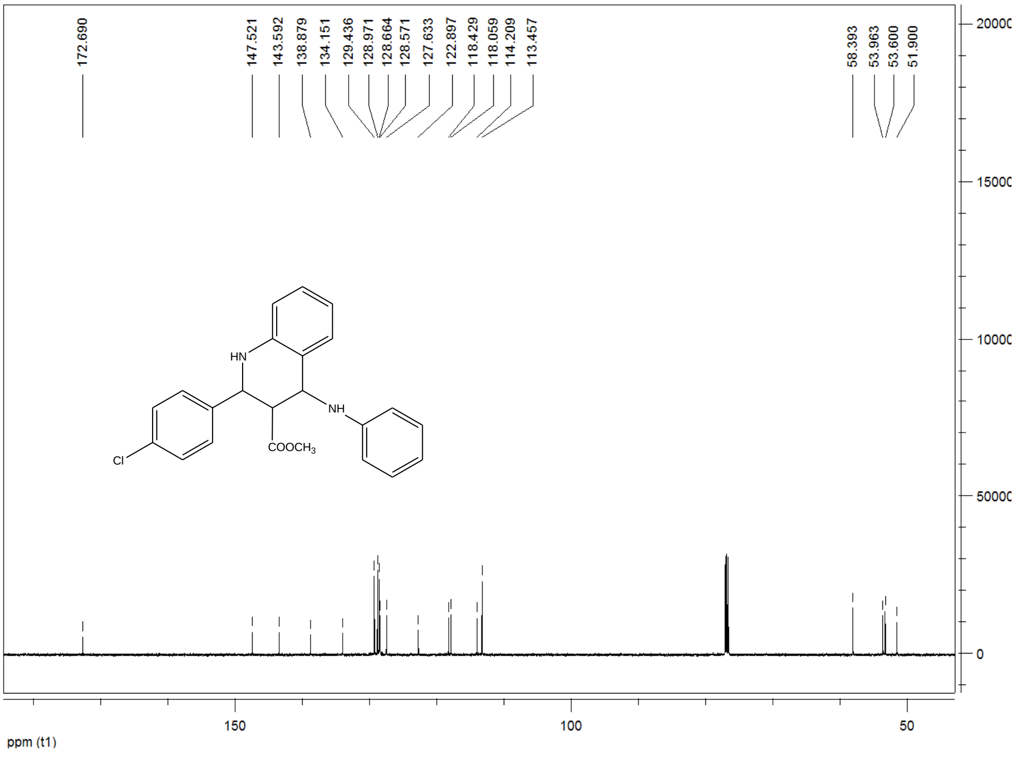

Methyl 6-methyl-4-(4-methylphenylamino)-2-phenyl-1,2,3,4-tetrahydroquinoline-3-carboxylate (1c):

Yellow solid, 63%, mp 164–166℃; ^{1}H NMR (600 MHz, $CDCl_3$) δ: 7.38 (brs, 2H, ArH), 7.32 (brs, 3H, ArH), 7.15 (s, 1H, ArH), 6.96 (d, J = 7.2 Hz, 2H, ArH), 6.89 (d, J = 7.8 Hz, 1H, ArH), 6.56 (d, J = 6.6 Hz, 2H, ArH), 6.49 (d, J = 7.8 Hz, 1H, ArH), 5.16 (d, J = 9.6 Hz, 1H, CH), 4.72 (d, J = 9.6 Hz, 1H, CH), 3.29 (s, 3H, OCH_3), 3.07 (t, J = 9.6 Hz, 1H, CH), 2.23 (s, 3H, CH_3), 2.18 (s, 3H, CH_3); ^{13}C NMR (150 MHz, $CDCl_3$) δ: 173.0, 145.3, 141.5, 140.6, 129.9, 129.1, 128.8, 128.4, 128.1, 127.4, 127.3, 127.0, 123.1, 114.3, 113.6, 59.0, 54.3, 53.8, 51.7, 20.7, 20.5; IR(KBr) υ: 3363, 3025, 2942, 2860, 1721, 1617, 1517, 1443, 1363, 1305, 1259, 1165, 1107, 996, 810, 771cm^{-1}; MS (m/z): 385.17 ([M+1]$^+$) 100%; HRMS (ESI) Calcd for $C_{25}H_{26}N_2NaO_2$ ([M+Na]$^+$): 409.1891. Found: 409.1889.

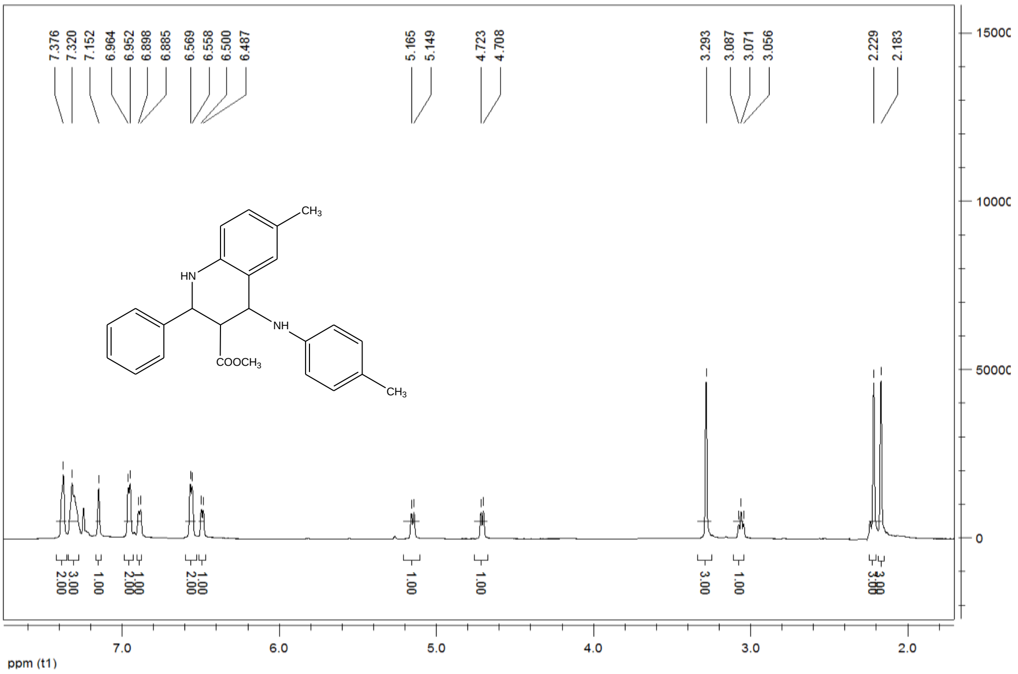

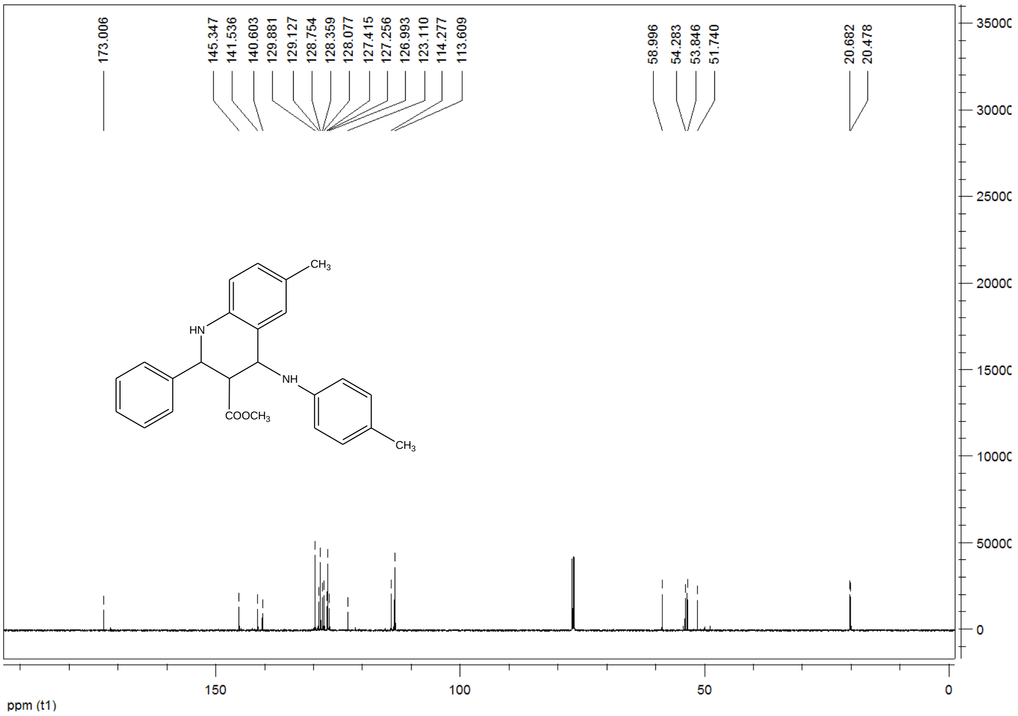

Methyl 2-(4-methoxyphenyl)-6-methyl-4-(4-methylphenylamino)-1,2,3,4-tetrahydro-quinoline-3-carboxylate (1d):

White solid, 46%, mp 179–181℃; ^{1}H NMR (600 MHz, $CDCl_3$) δ: 7.30 (d, *J* = 8.4 Hz, 2H, ArH), 7.15 (s, 1H, ArH), 6.96 (d, *J* = 8.4 Hz, 2H, ArH), 6.88–6.84 (m, 3H, ArH), 6.59 (d, *J* = 7.8 Hz, 2H, ArH), 6.47 (d, *J* = 7.8 Hz, 1H, ArH), 5.14 (d, *J* = 9.6 Hz, 1H, CH), 4.64 (d, *J* = 9.6 Hz, 1H, CH), 3.79 (s, 3H, OCH_3), 3.29 (s, 3H, OCH_3), 3.01 (t, *J* = 9.6 Hz, 1H, CH), 2.23 (s, 3H, CH_3), 2.18 (s, 3H, CH_3); ^{13}C NMR (150 MHz, $CDCl_3$) δ: 173.1, 159.5, 145.4, 141.6, 132.5, 129.9, 129.0, 128.4, 127.9, 127.3, 127.0, 123.2, 114.2, 114.0, 113.9, 113.6, 58.6, 55.3, 54.5, 54.2, 51.7, 20.6, 20.4; IR(KBr) υ: 3382, 3014, 2946, 1721, 1618, 1508, 1435, 1357, 1298, 1244, 1168, 1026, 804cm^{-1}; MS (*m/z*): 413.61 ([M-1]$^+$) 100%; HRMS (ESI) Calcd for $C_{26}H_{28}N_2NaO_3$ ([M+Na]$^+$): 439.1997. Found: 439.1995.

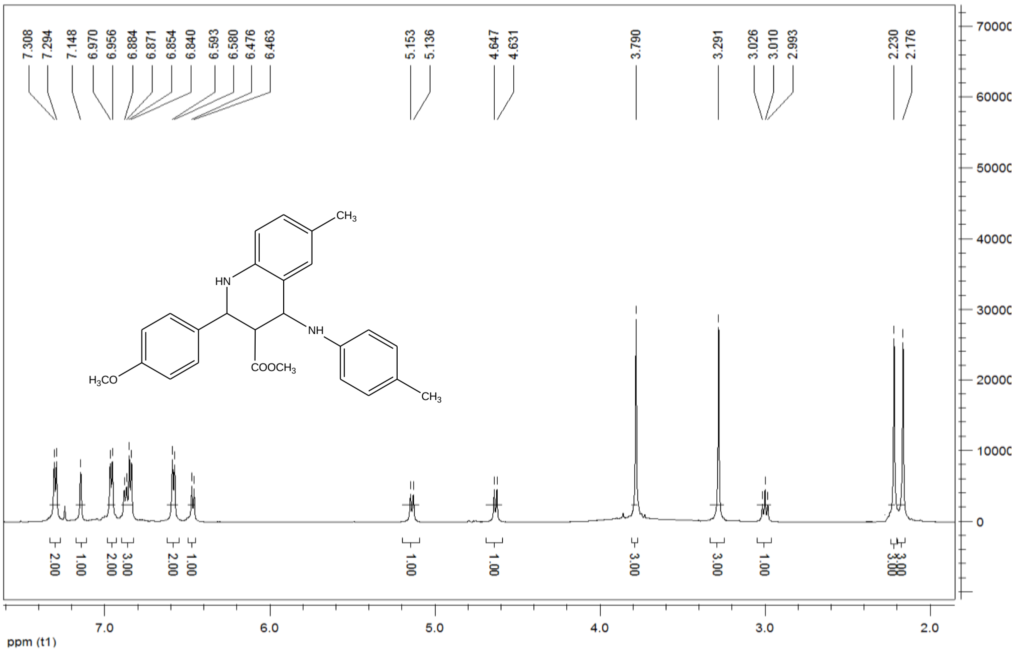

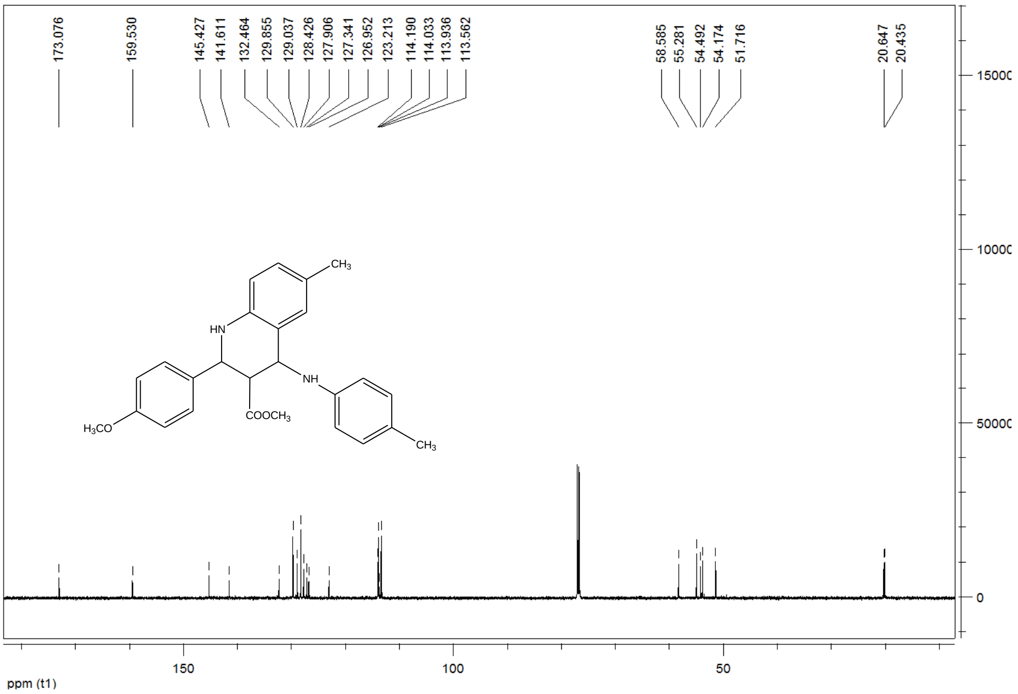

Methyl 2-(4-isopropylphenyl)-6-methyl-4-(4-methylphenylamino)-1,2,3,4-tetrahydro-quinoline-3-carboxylate (1e):

White solid, 41%, mp 147–149℃; ^{1}H NMR (600 MHz, $CDCl_3$) δ: 7.29 (d, *J* = 8.4 Hz, 2H, ArH), 7.18 (d, *J* = 8.4 Hz, 2H, ArH), 7.14 (s, 1H, ArH), 6.95 (d, *J* = 7.8 Hz, 2H, ArH), 6.89 (d, *J* = 7.8 Hz, 1H, ArH), 6.54 (d, *J* = 8.4 Hz, 2H, ArH), 6.48 (d, *J* = 8.4 Hz, 1H, ArH), 5.14 (d, *J* = 9.6 Hz, 1H, CH), 4.70 (d, *J* = 9.6 Hz, 1H, CH), 3.31 (s, 3H, OCH_3), 3.07 (t, *J* = 9.6 Hz, 1H, CH), 2.91–2.88 (m, 1H, CH), 2.23 (s, 3H, CH_3), 2.18 (s, 3H, CH_3), 1.23 (d, *J* = 6.6 Hz, 6H, CH_3); ^{13}C NMR (150 MHz, $CDCl_3$) δ: 173.0, 149.0, 145.3, 141.5, 138.0, 129.8, 129.1, 128.2, 127.3, 127.1, 127.0, 126.8, 123.0, 114.2, 113.6, 58.5, 54.2, 53.5, 51.7, 33.9, 24.0, 23.9, 20.6, 20.4; IR(KBr) υ: 3368, 3022, 2958, 1720, 1620, 1515, 1442, 1360, 1305, 1165, 1097, 995, 809cm^{-1}; MS (*m/z*): 427.99 ([M+1]$^{+}$) 100%; HRMS (ESI) Calcd for $C_{28}H_{32}N_2NaO_2$ ([M+Na]$^{+}$): 451.2361. Found: 451.2361.

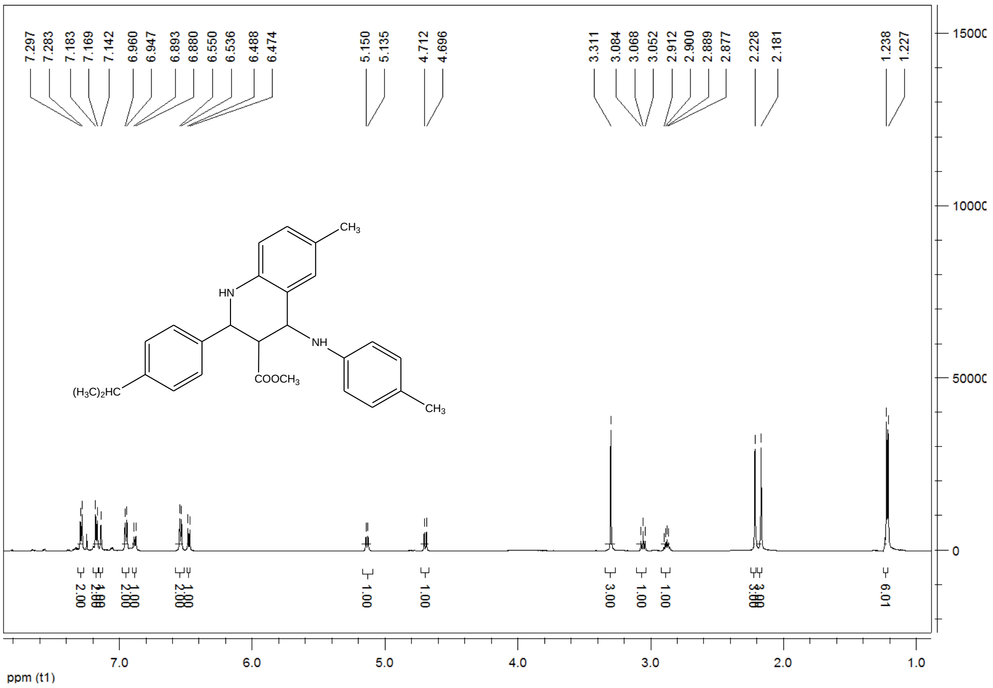

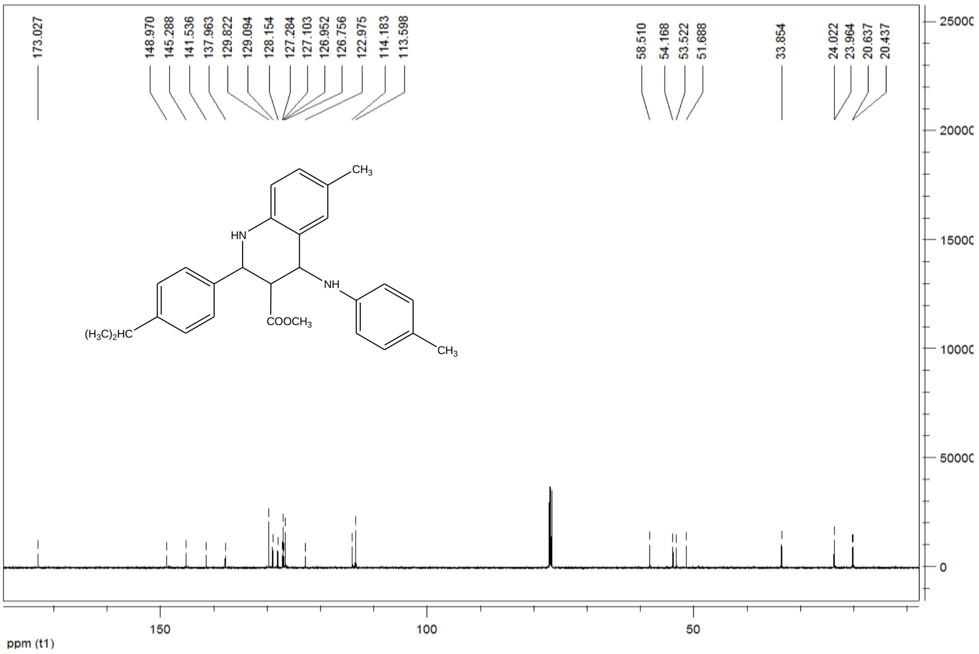

Methyl 2-(4-chlorophenyl)-6-methyl-4-(4-methylphenylamino)-1,2,3,4-tetrahydroquinoline-3-carboxylate (1f):

White solid, 48%, mp 154–155℃; ^{1}H NMR (600 MHz, $CDCl_3$) δ : 7.33–7.28(m, 4H, ArH), 7.15 (s, 1H, ArH), 6.96 (d, *J* = 7.8 Hz, 2H, ArH), 6.89 (d, *J* = 8.4 Hz, 1H, ArH), 6.57 (d, *J* = 7.8 Hz, 2H, ArH), 6.49 (d, *J* = 7.8 Hz, 1H, ArH), 5.14 (d, *J* = 9.6 Hz, 1H, CH), 4.68 (d, *J* = 9.6 Hz, 1H, CH), 3.30 (s, 3H, OCH_3), 3.02 (t, *J* = 9.6 Hz, 1H, CH), 2.23 (s, 3H, CH_3), 2.18 (s, 3H, CH_3); ^{13}C NMR (150 MHz, $CDCl_3$) δ : 172.7, 145.1, 141.2, 139.1, 134.0, 130.2, 129.8, 129.1, 128.9, 128.6, 128.0, 127.1, 123.1, 115.3, 114.3, 113.5, 58.4, 54.3, 53.8, 51.8, 20.6, 20.4; IR(KBr) υ : 3392, 3020, 2947, 2915, 1773, 1669, 1616, 1525, 1503, 1434, 1360, 1300, 1264, 1245, 1221, 1197, 1121, 1088, 1049, 1041, 995, 879, 808cm^{-1}; HRMS (ESI) Calcd for $C_{25}H_{25}ClN_2NaO_2$ ($[M+Na]^+$): 443.1502. Found: 443.1495.

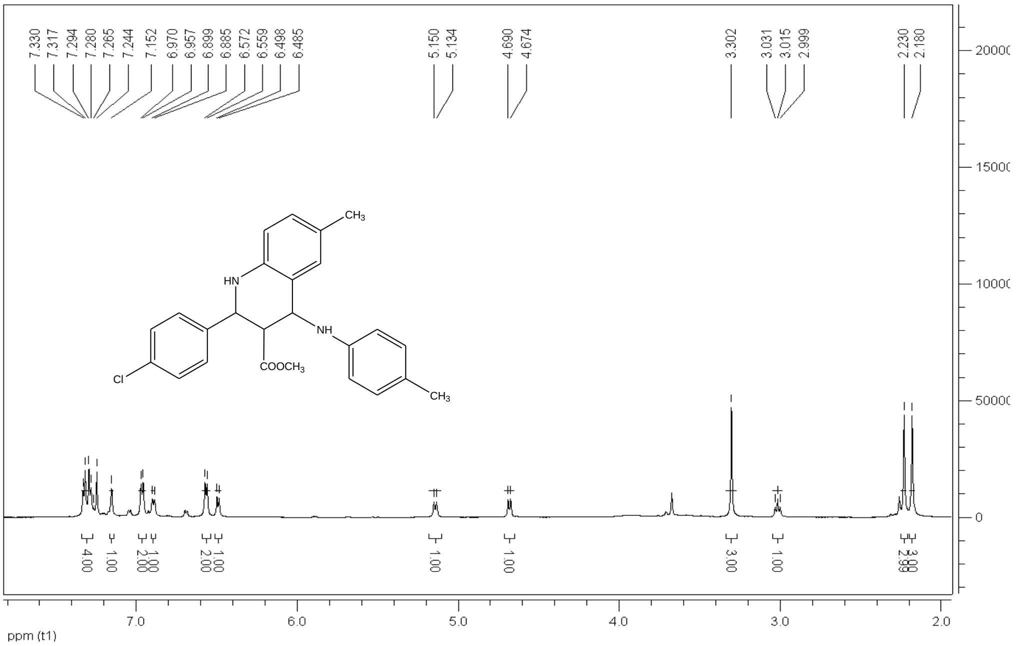

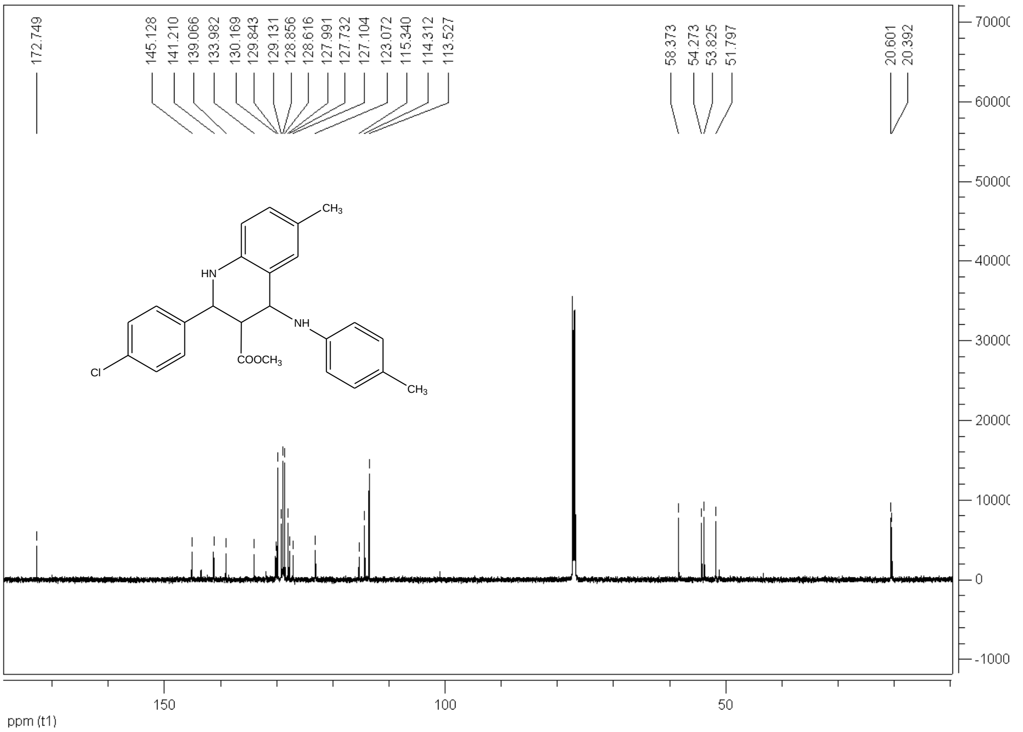

Methyl 6-methoxy-4-(4-methoxyphenylamino)-2-(4-methylphenyl)-1,2,3,4-tetrahydro-quinoline-3-carboxylate (1g):

White solid, 51%, mp 160–162℃; ^{1}H NMR (600 MHz, $CDCl_3$) δ: 7.27 (d, *J* = 7.8 Hz, 2H, ArH), 7.12 (d, *J* = 7.8 Hz, 2H, ArH), 6.99 (brs, 1H, ArH), 6.75–6.73 (m, 2H, ArH), 6.70–6.68 (m, 1H, ArH), 6.64 (d, *J* = 9.0 Hz, 2H, ArH), 6.52 (d, *J* = 9.0 Hz, 1H, ArH), 5.11 (d, *J* = 9.6 Hz, 1H, CH), 4.61 (d, *J* = 9.6 Hz, 1H, CH), 3.74 (s, 3H, OCH_3), 3.66 (s, 3H, OCH_3), 3.29 (s, 3H, OCH_3), 3.06 (t, *J* = 9.6 Hz, 1H, CH), 2.33 (s, 3H, CH_3); ^{13}C NMR (150 MHz, $CDCl_3$) δ: 173.1, 152.4, 141.7, 138.0, 137.4, 129.3, 127.1, 124.5, 115.3, 115.2, 114.9, 112.6, 59.1, 55.7, 55.5, 54.0, 51.7, 21.2; IR(KBr) υ: 3374, 2832, 1722, 1507, 1457, 1335, 1244, 1170, 1101, 1034, 815cm^{-1}; MS (*m/z*): 429.37 ([M-1]$^{+}$) 100%; HRMS (ESI) Calcd for $C_{26}H_{28}N_2NaO_4$ ([M+Na]$^{+}$): 455.1946. Found: 455.1891.

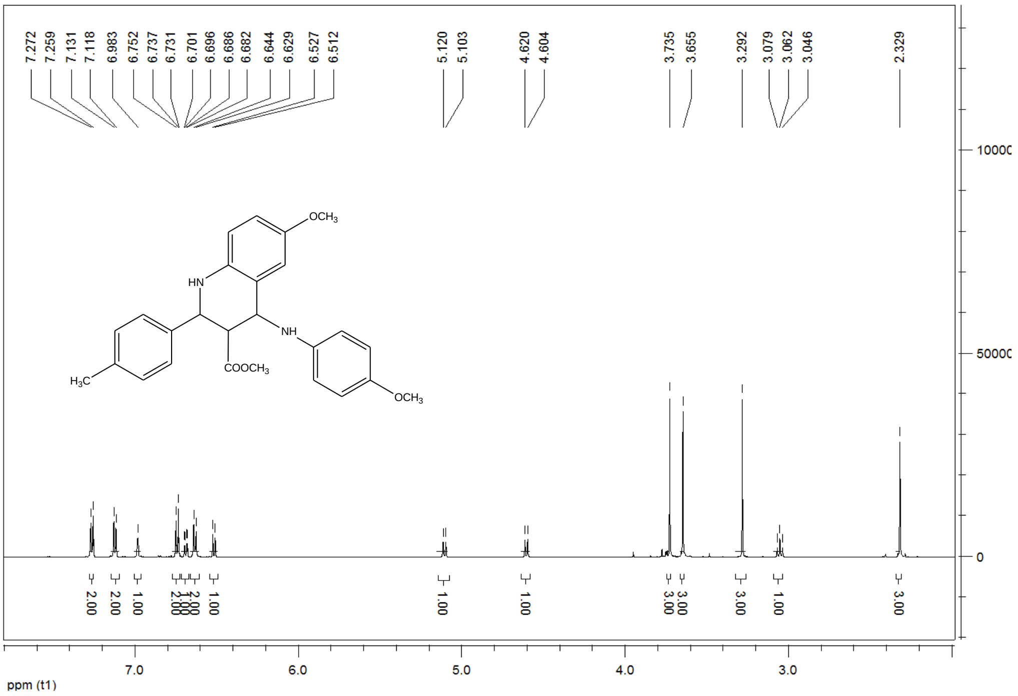

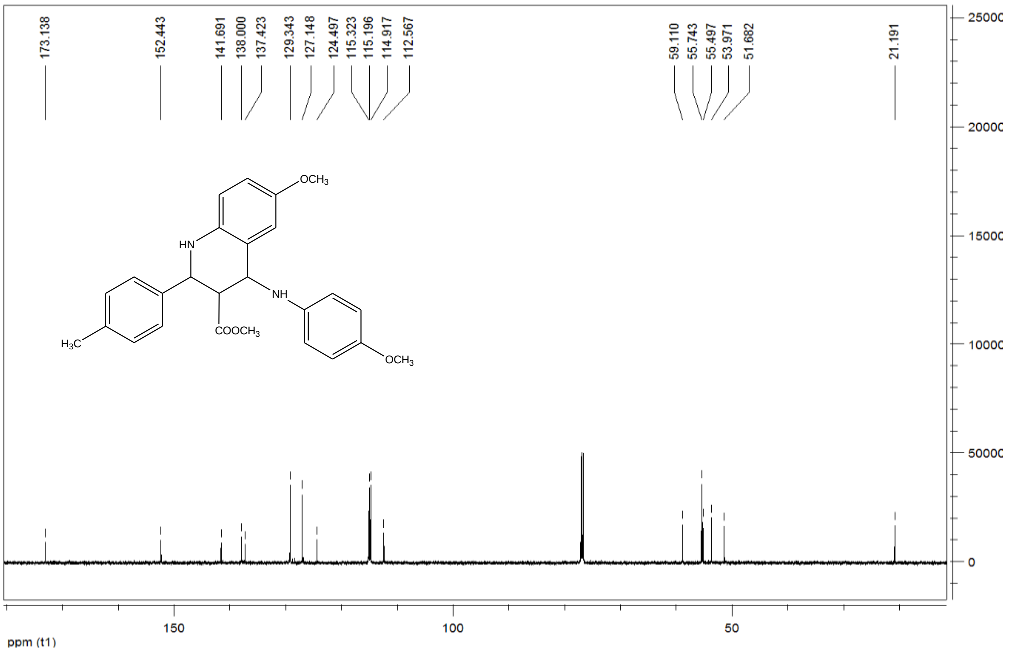

Methyl 6-chloro-4-(4-chlorophenylamino)-2-phenyl-1,2,3,4-tetrahydroquinoline-3-carboxylate (1h):

White solid, 59%, mp 190–192℃; ^{1}H NMR (600 MHz, $CDCl_3$) δ: 7.37–7.31 (m, 6H, ArH), 7.09 (d, *J* = 8.4 Hz, 2H, ArH), 7.03 (d, *J* = 9.0 Hz, 1H, ArH), 6.53 (d, *J* = 8.4 Hz, 2H, ArH), 6.50 (d, *J* = 8.4 Hz, 1H, ArH), 5.11 (d, *J* = 9.0 Hz, 1H, CH), 4.72 (d, *J* = 9.6 Hz, 1H, CH), 3.31 (s, 3H, OCH_3), 3.04 (t, *J* = 9.6 Hz, 1H, CH); ^{13}C NMR (150 MHz, $CDCl_3$) δ: 172.4, 145.5, 142.3, 139.8, 129.3, 128.9, 128.7, 128.6, 127.4, 127.1, 123.6, 122.8, 122.7, 115.4, 114.6, 58.6, 53.8, 52.7, 51.9; IR(KBr) υ: 3410, 3361, 3032, 2950, 1719, 1598, 1490, 1430, 1361, 1290, 1248, 1170, 1088, 992, 900, 813, 765cm^{-1}; HRMS (ESI) Calcd for $C_{23}H_{20}Cl_2N_2NaO_2$ ($[M+Na]^+$): 449.0799. Found: 449.0796.

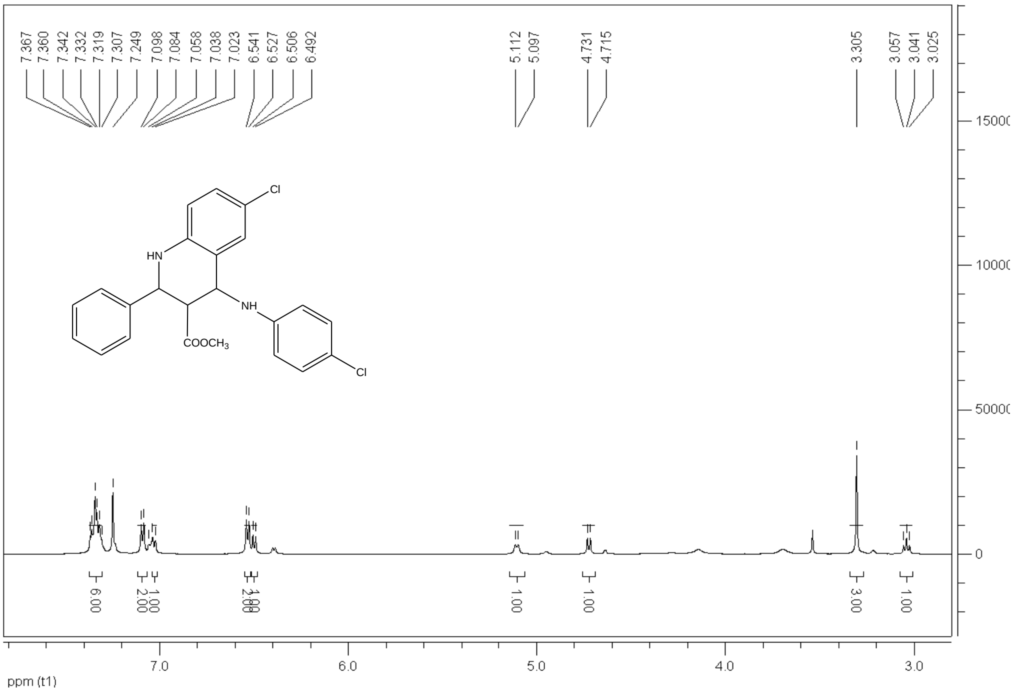

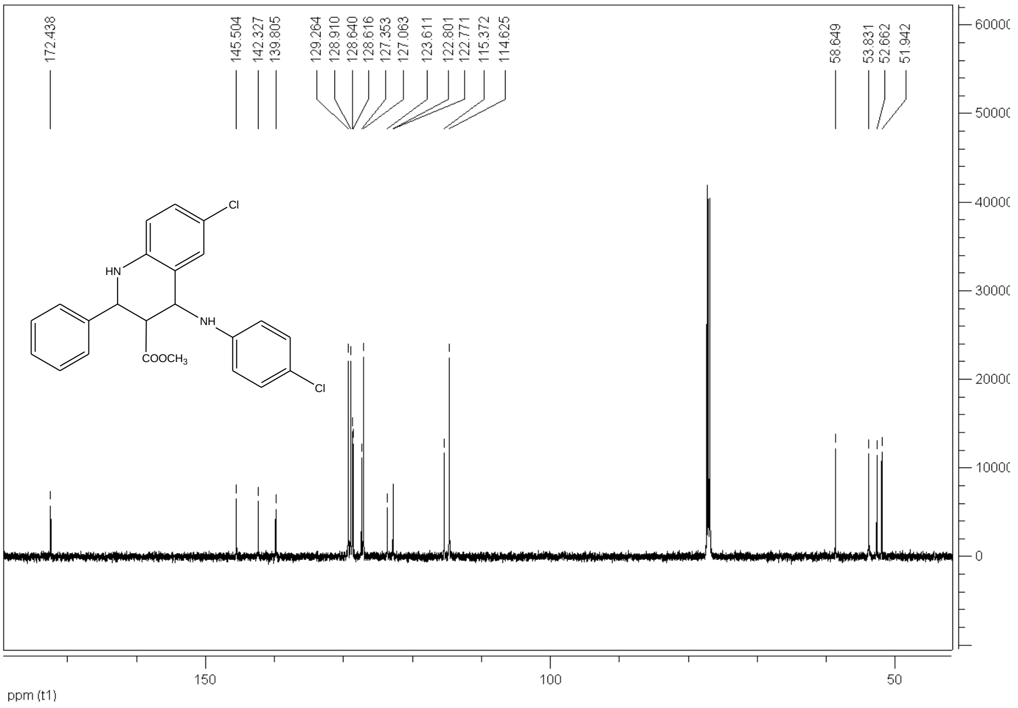

Methyl 4-(4-chlorophenylamino)-2-(4-methylphenyl)-6-chloro-1,2,3,4-tetrahydroquinoline-3-carboxylate (1i):

White solid, 66%, mp 211–213℃; ^{1}H NMR (600 MHz, $CDCl_3$) δ: 7.25–7.23 (m, 3H, ArH), 7.14–7.09 (m, 4H, ArH), 7.03–7.01 (m, 1H, ArH), 6.56 (d, J = 9.0 Hz, 2H, ArH), 6.49 (d, J = 9.0 Hz, 1H, ArH), 5.10 (d, J = 9.6 Hz, 1H, CH), 4.68 (d, J = 9.6 Hz, 1H, CH), 3.31 (s, 3H, OCH_3), 3.03 (t, J = 9.6 Hz, 1H, CH), 2.33 (s, 3H, CH_3); ^{13}C NMR (150 MHz, $CDCl_3$) δ: 172.5, 145.6, 142.4, 138.4, 136.7, 129.5, 129.2, 128.5, 127.2, 127.0, 123.7, 122.7, 122.6, 115.3, 114.6, 58.5, 54.0, 52.8, 51.9, 21.2; IR(KBr) υ: 3370, 2947, 1720, 1598, 1488, 1434, 1354, 1292, 1248, 1172, 1091, 898, 814cm^{-1}; MS (m/z): 439.35 ($[M+1]^+$) 100%; HRMS (ESI) Calcd for $C_{24}H_{22}Cl_2N_2NaO_2$ ($[M+Na]^+$): 463.0955. Found: 463.0953.

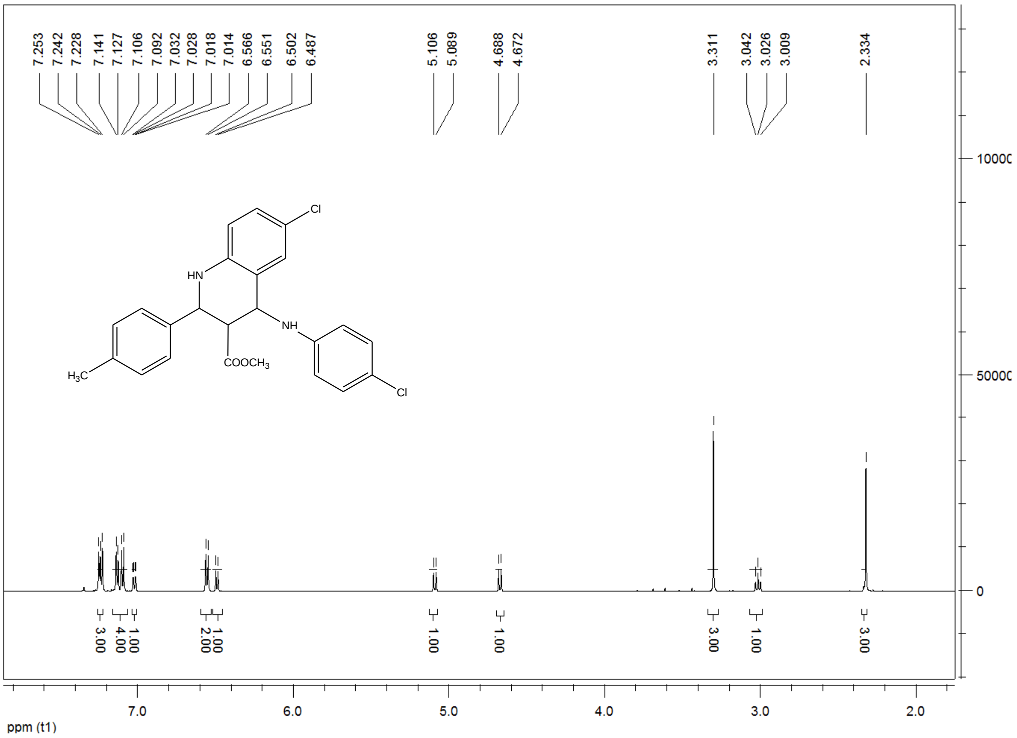

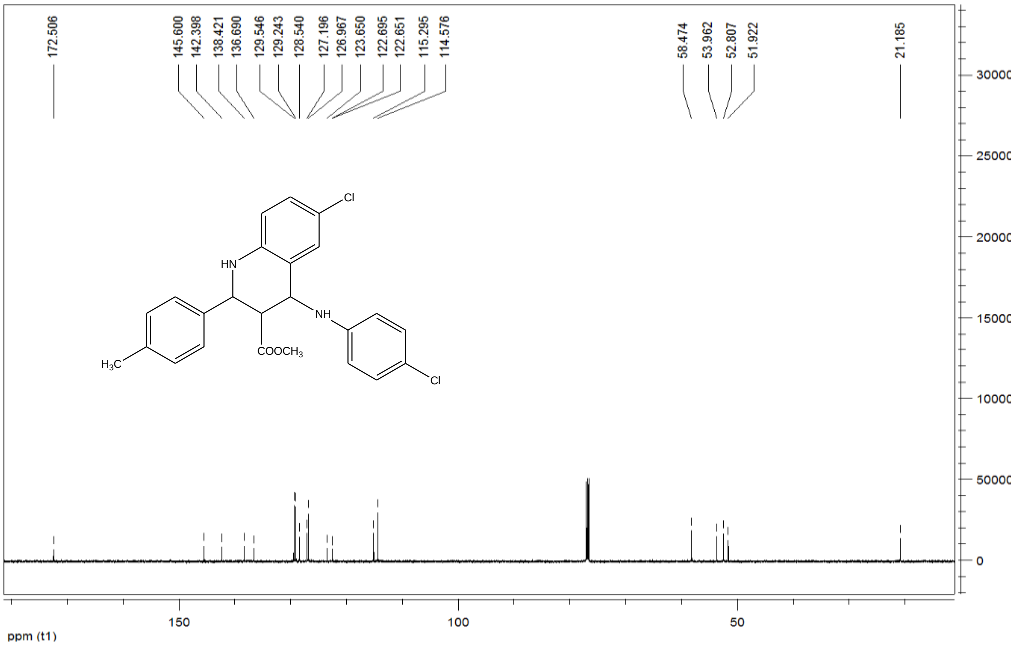

Methyl 6-chloro-4-(4-chlorophenylamino)-2-(4-methoxyphenyl)-1,2,3,4-tetrahydroquinoline-3-carboxylate (1j):

White solid, 54%, mp 186–188℃; ^{1}H NMR (600 MHz, $CDCl_3$) δ: 7.27 (d, *J* = 8.4 Hz, 2H, ArH), 7.25 (s, 1H, ArH), 7.10 (d, *J* = 9.0 Hz, 2H, ArH), 7.02 (d, *J* = 7.2 Hz, 1H, ArH), 6.86 (d, *J* = 8.4 Hz, 2H, ArH), 6.57 (d, *J* = 8.4 Hz, 2H, ArH), 6.48 (d, *J* = 8.4 Hz, 1H, ArH), 5.10 (d, *J* = 9.6 Hz, 1H, CH), 4.65 (d, *J* = 9.6 Hz, 1H, CH), 3.80 (s, 3H, OCH_3), 3.30 (s, 3H, OCH_3), 2.99 (t, *J* = 9.6 Hz, 1H, CH); ^{13}C NMR (150 MHz, $CDCl_3$) δ: 172.5, 159.7, 145.6, 142.4, 131.6, 129.2, 128.5, 128.3, 127.2, 123.7, 122.8, 115.3, 114.6, 114.2, 58.3, 55.3, 54.1, 53.0 51.9; IR(KBr) υ: 3373, 2949, 2837, 1721, 1599, 1515, 1488, 1432, 1353, 1285, 1247, 1198, 1176, 1087, 1032, 995, 896, 815cm^{-1}; HRMS (ESI) Calcd for $C_{24}H_{22}Cl_2N_2NaO_3$ ($[M+Na]^+$): 479.0904. Found: 479.0900.

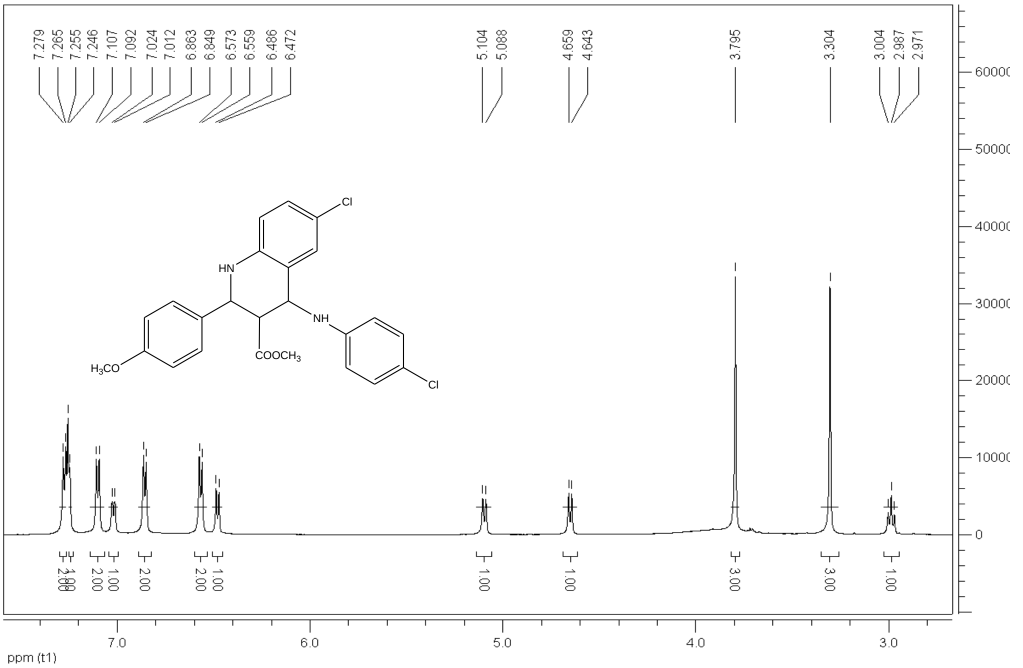

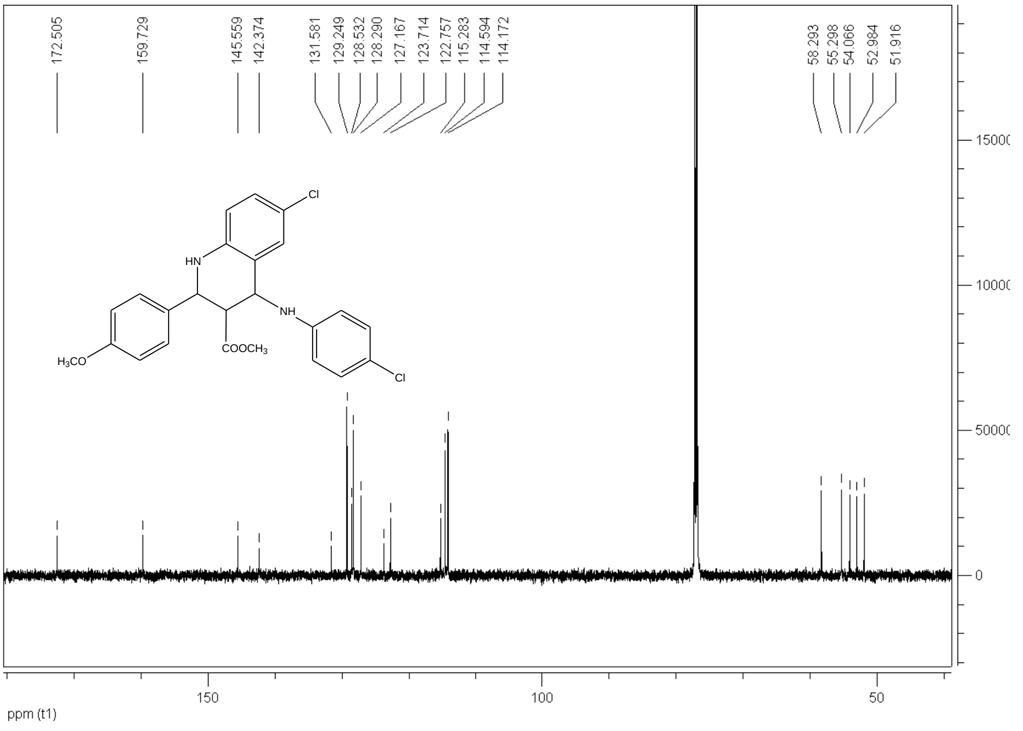

Methyl 6-chloro-2-(4-chlorophenyl)-4-(4-chlorophenylamino)-1,2,3,4-tetrahydroquinoline-3-carboxylate (1k):

White solid, 61%, mp 195–196℃; ^{1}H NMR (600 MHz, $CDCl_3$) δ : 7.31 (s, 5H, ArH), 7.11 (d, *J* = 8.4 Hz, 2H, ArH), 7.04 (d, *J* = 8.4 Hz, 1H, ArH), 6.56 (d, *J* = 8.4 Hz, 2H, ArH), 6.51 (d, *J* = 8.4 Hz, 1H, ArH), 5.11 (d, *J* = 9.6 Hz, 1H, CH), 4.71 (d, *J* = 9.6 Hz, 1H, CH), 3.32 (s, 3H, OCH_3), 3.00 (t, *J* = 9.6 Hz, 1H, CH); ^{13}C NMR (150 MHz, $CDCl_3$) δ : 172.2, 145.4, 142.1, 138.3, 134.4, 129.3, 129.0, 128.7, 128.5, 127.3, 123.1, 122.9, 115.5, 114.6, 58.1, 53.9, 52.7, 52.0; IR(KBr) υ : 3382, 3030, 2946, 2878, 1877, 1722, 1670, 1598, 1488, 1433, 1352, 1290, 1250, 1196, 1172, 1090, 995, 946, 896, 815, 753cm^{-1}; HRMS (ESI) Calcd for $C_{23}H_{19}Cl_3N_2NaO_2$ ($[M+Na]^+$): 483.0409. Found: 483.0406.

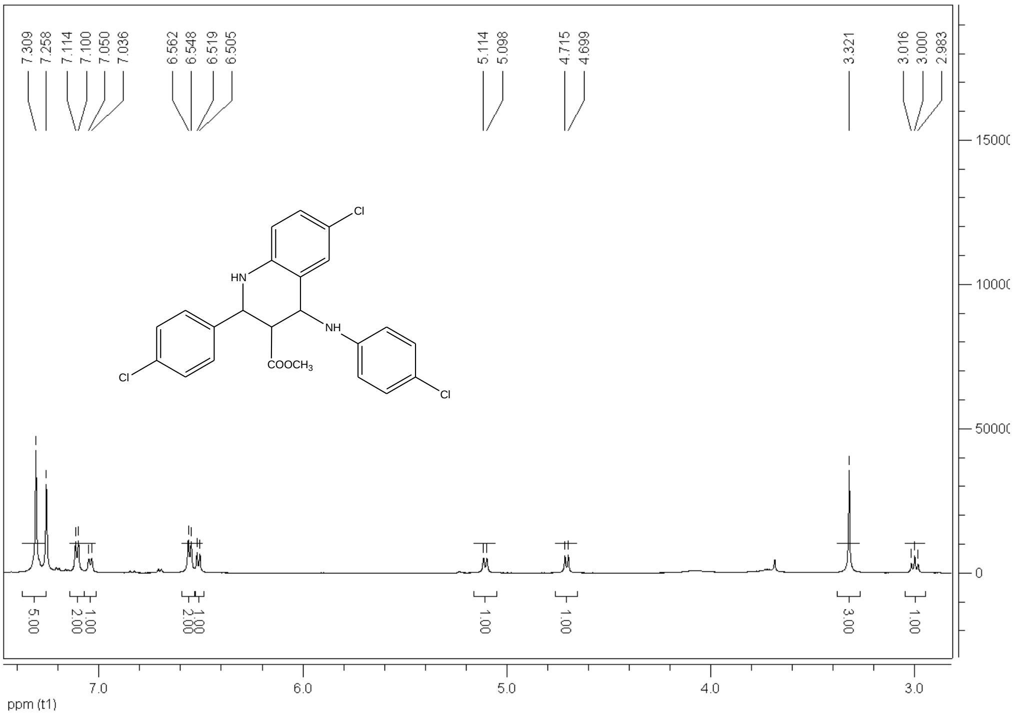

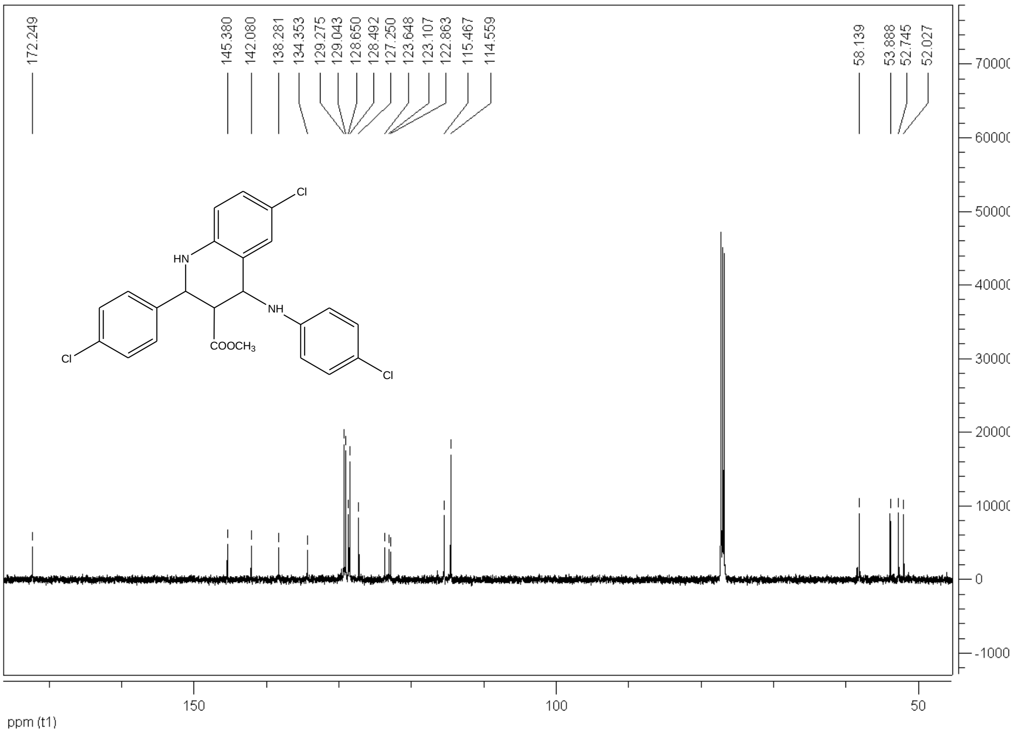

Methyl 6-bromo-4-(4-bromophenylamino)-2-phenyl-1,2,3,4-tetrahydroquinoline-3-carboxylate (1l):

Yellow solid, 67%, mp 156–157℃; ^{1}H NMR (600 MHz, DMSO) δ: 7.39–7.32(m, 5H, ArH), 7.21(d, *J*=8.4 Hz, 2H, ArH), 7.12–7.09(m, 2H, ArH, NH), 6.64–6.62(m, 3H, ArH, NH), 6.55(s, 1H, ArH), 6.31(d, 1H, ArH), 5.11(t, 1H, CH), 4.59(d, 1H, CH), 3.17(s, 3H, OCH_3), 2.92(t, 1H, CH); ^{13}C NMR (150 MHz, DMSO) δ: 172.1, 147.5, 144.1, 140.1, 131.5, 130.2, 128.7, 128.2, 127.3, 124.1, 115.8, 114.4, 106.7, 58.5, 52.5, 52.2, 51.2; IR(KBr) υ: 3362, 2845, 1727, 1596, 1546, 1487, 1433, 1336, 1325, 1245, 1160, 760cm^{-1}; MS (*m/z*): 512.46 ([M+1]$^{+}$) 100%; MS (*m/z*): 512.46 ([M+1]$^{+}$) 100%; HRMS (ESI) Calcd for $C_{23}H_{20}Br_2N_2NaO_2$ ([M+Na]$^{+}$): 536.9789. Found: 536.9783.

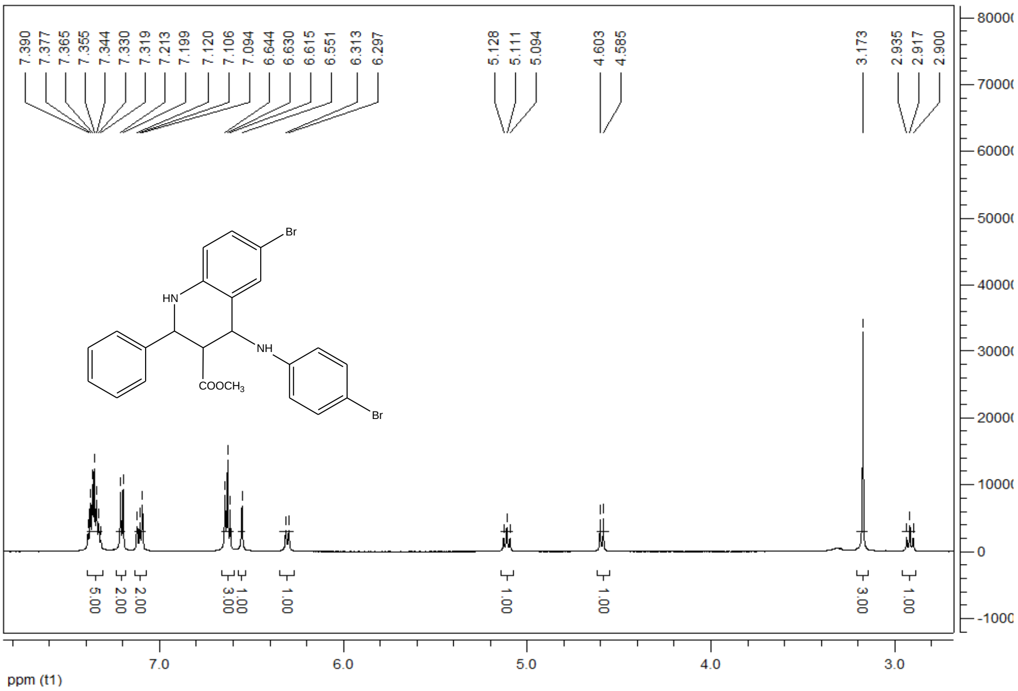

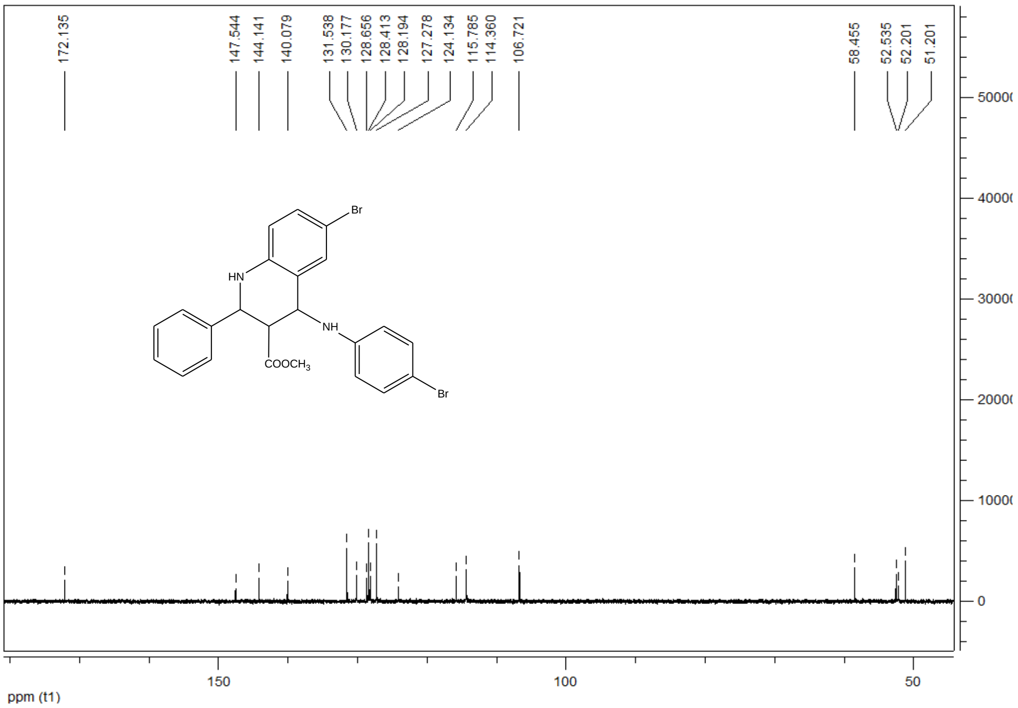

Methyl 6-bromo-4-(4-bromophenylamino)-2-(4-methylphenyl)-1,2,3,4-tetrahydroquinoline-3-carboxylate (1m):

White solid, 56%, mp 214–216℃; ^{1}H NMR (600 MHz, $CDCl_3$) δ: 7.37 (s, 1H, ArH), 7.23 (d, *J* = 6.6 Hz, 4H, ArH), 7.15 (t, *J* = 7.8 Hz, 3H, ArH), 6.55 (d, *J* = 8.4 Hz, 2H, ArH), 6.44 (d, *J* = 8.4 Hz, 1H, ArH), 5.10 (d, *J* = 9.0 Hz, 1H, CH), 4.69 (d, *J* = 9.6 Hz, 1H, CH), 3.32 (s, 3H, OCH_3), 3.01 (t, *J* = 9.6 Hz, 1H, CH); ^{13}C NMR (150 MHz, $CDCl_3$) δ: 172.4, 145.9, 142.8, 138.4, 136.6, 132.1, 131.4, 130.1, 129.5, 126.9, 123.9, 115.7, 115.0, 109.8, 109.7, 58.3, 53.7, 52.6, 51.9, 21.2; IR(KBr) υ: 3376, 3025, 2947, 1720, 1596, 1515, 1485, 1430, 1347, 1289, 1247, 1198, 1169, 1072, 996, 895cm^{-1}; HRMS (ESI) Calcd for $C_{24}H_{22}Br_2N_2NaO_2$ ([M+Na]$^+$): 552.9925. Found: 552.9916.

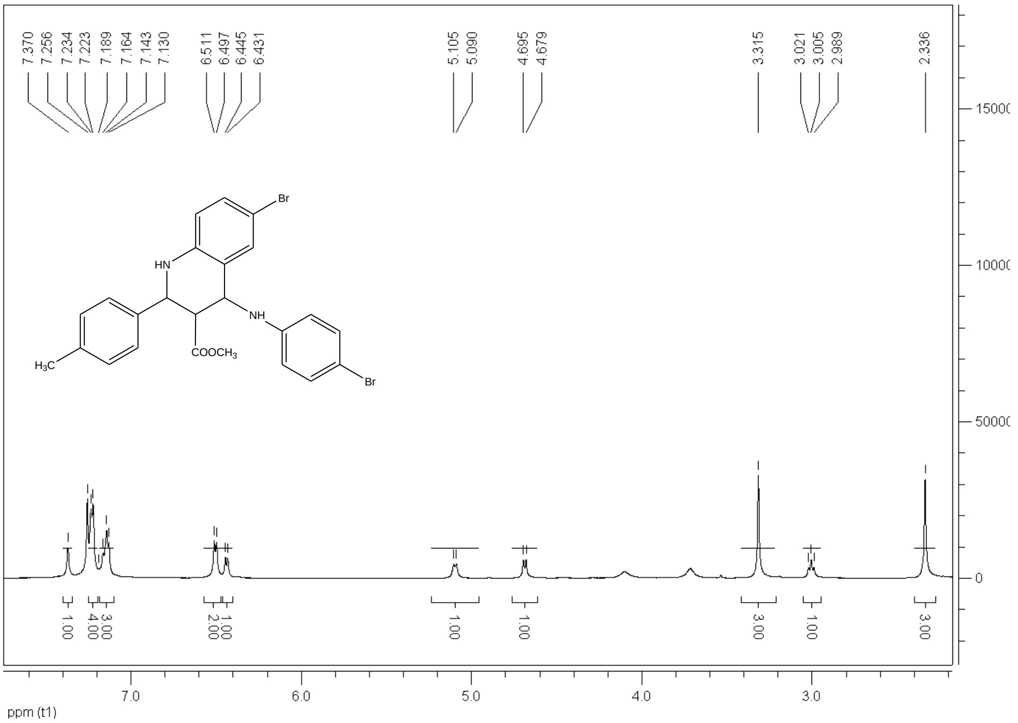

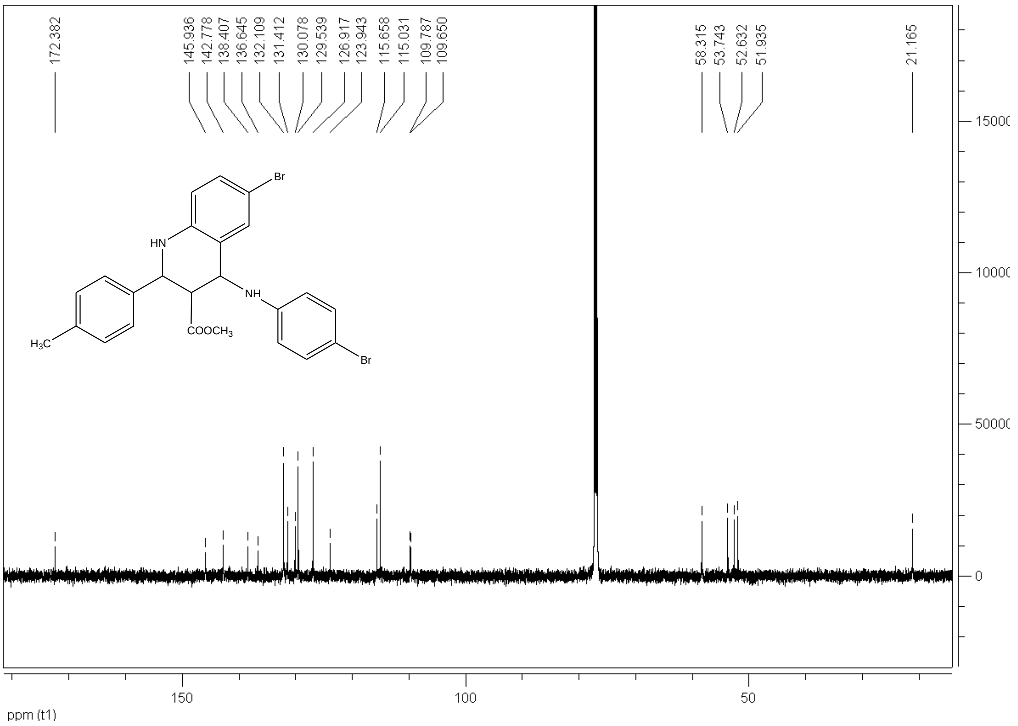

Methyl 6-chloro-2-(4-chlorophenyl)-4-(4-methylphenylamino)-1,2,3,4-tetrahydroquinoline-3-carboxylate (2a):

Orange yellow solid, 60%, mp 191–193℃; ^{1}H NMR (600 MHz, $CDCl_3$) δ: 7.33–7.29 (m, 4H, ArH), 7.09 (t, *J* = 8.4 Hz, 3H, ArH), 6.91 (d, *J* = 7.2 Hz, 1H, ArH), 6.56 (d, *J* = 9.0 Hz, 2H, ArH), 6.51 (d, *J* = 7.8 Hz, 1H, ArH), 5.14 (d, *J* = 10.2 Hz, 1H, CH), 4.68 (d, *J* = 9.6 Hz, 1H, CH), 3.32 (s, 3H, OCH_3), 3.02 (t, *J* = 9.6 Hz, 1H, CH), 2.19 (s, 3H, CH_3); ^{13}C NMR (150 MHz, $CDCl_3$) δ: 172.6, 145.9, 141.2, 138.8, 134.1, 129.4, 129.2, 128.9, 128.6, 127.9, 127.8, 122.4, 122.2, 114.5, 114.4, 58.3, 54.0, 53.5, 51.9, 20.6; IR(KBr) υ: 3373, 3025, 2947, 1723, 1596, 1505, 1432, 1361, 1292, 1261, 1195, 1158, 1089, 1015, 991, 961, 877, 813cm^{-1}; HRMS (ESI) Calcd for $C_{24}H_{22}Cl_2N_2NaO_2$ ($[M+Na]^+$): 463.0955. Found: 463.0951.

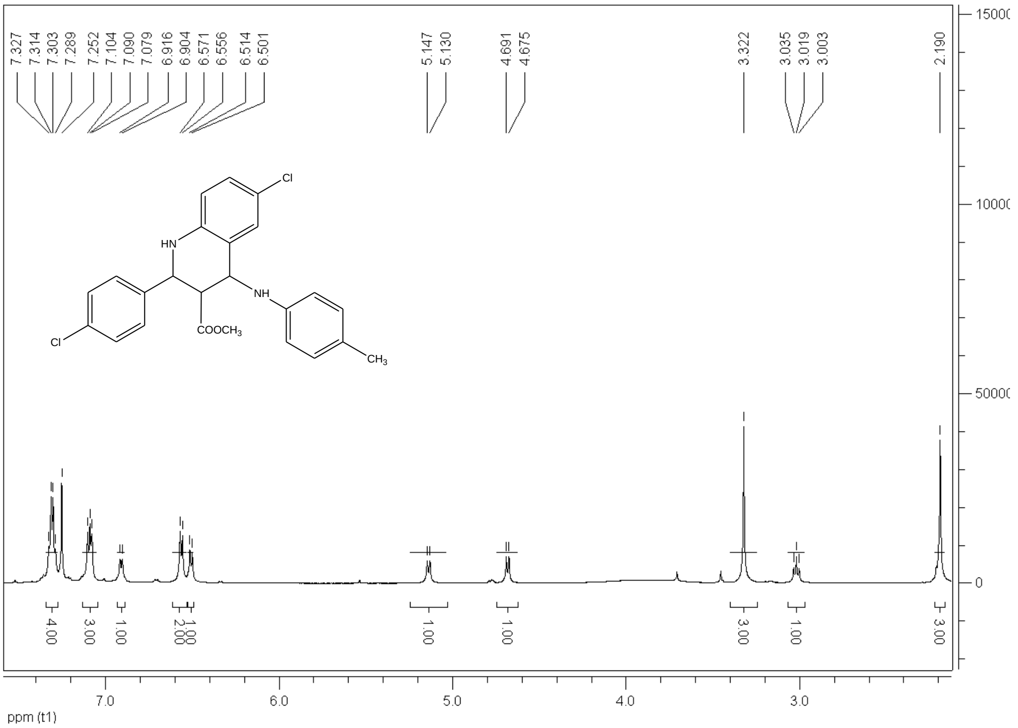

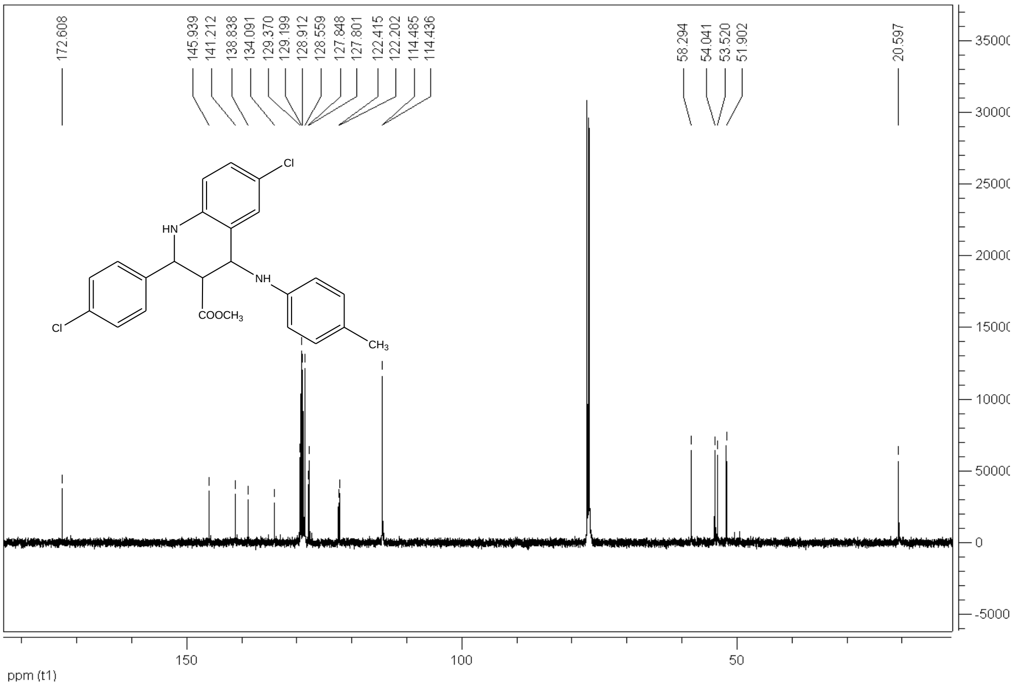

Methyl 6-chloro-2-(4-chlorophenyl)-4-(4-methoxyphenylamino)-1,2,3,4-tetrahydroquinoline-3-carboxylate (2b):

White solid, 63%, mp 182–184℃; ^{1}H NMR (600 MHz, $CDCl_3$) δ: 7.33–7.29 (m, 4H, ArH), 7.09 (d, *J* = 8.4 Hz, 2H, ArH), 6.87 (s, 1H, ArH), 6.71 (d, *J* = 6.6 Hz, 1H, ArH), 6.57–6.53 (m, 3H, ArH), 5.15 (d, *J* = 9.0 Hz, 1H, CH), 4.64 (d, *J* = 9.6 Hz, 1H, CH), 3.66 (s, 3H, OCH_3), 3.32 (s, 3H, OCH_3), 3.03 (t, *J* = 9.6 Hz, 1H, CH); ^{13}C NMR (150 MHz, $CDCl_3$) δ: 172.6, 152.8, 145.8, 138.8, 137.6, 134.1, 129.2, 128.9, 128.6, 123.5, 122.6, 115.6, 115.1, 114.6, 112.5, 58.6, 55.7, 54.8, 53.5, 51.9; IR(KBr) υ: 3384, 2949, 1723, 1642, 1599, 1499, 1355, 1290, 1199, 1165, 1091, 1041, 957, 882, 811cm^{-1}; HRMS (ESI) Calcd for $C_{24}H_{22}Cl_2N_2NaO_3$ ($[M+Na]^+$): 479.0904. Found: 479.0898.

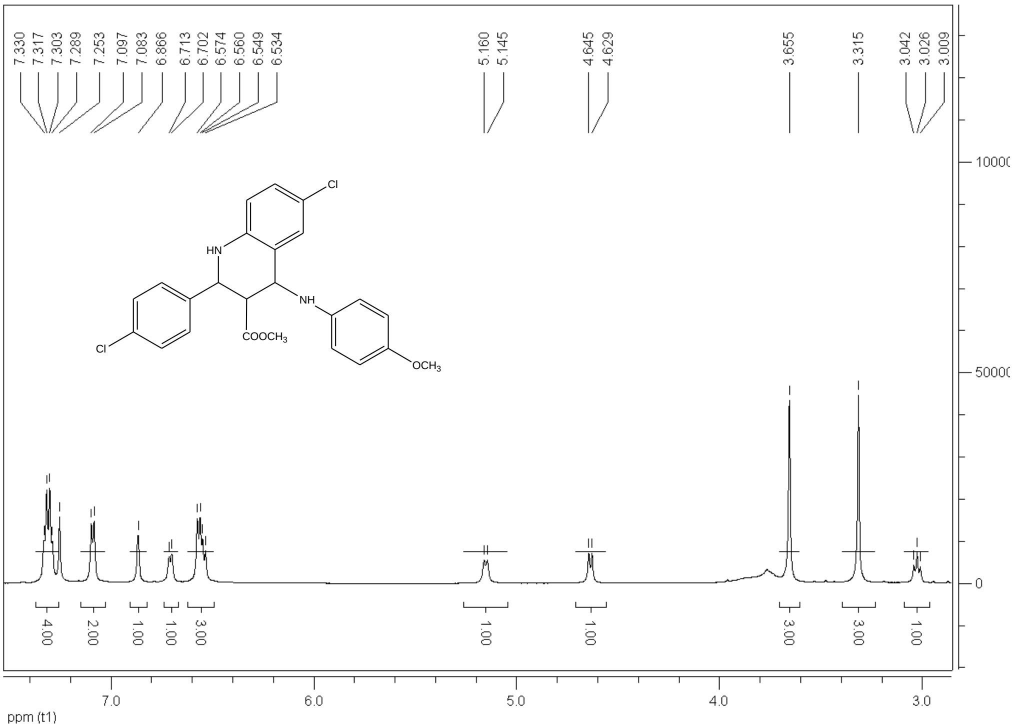

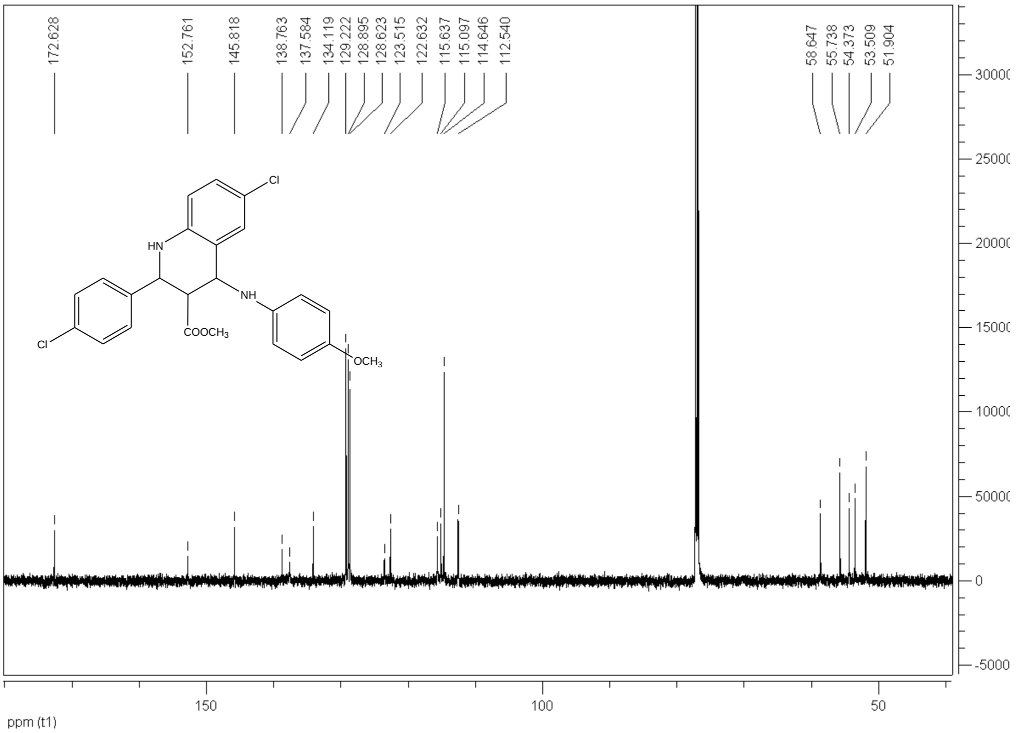

Methyl 6-chloro-4-(4-methylphenylamino)-2-phenyl-1,2,3,4-tetrahydroquinoline-3-carboxylate (2c):

White solid, 56%, mp 168–170℃; ^{1}H NMR (600 MHz, $CDCl_3$) δ: 7.37 (d, *J* = 6.6 Hz, 2H, ArH), 7.34–7.29 (m, 3H, ArH), 7.09 (d, *J* = 9.0 Hz, 3H, ArH), 6.90 (d, *J* = 7.8 Hz, 1H, ArH), 6.55 (d, *J* = 8.4 Hz, 2H, ArH), 6.50 (d, *J* = 7.8 Hz, 1H, ArH), 5.15 (d, *J* = 9.6 Hz, 1H, CH), 4.71 (d, *J* = 9.6 Hz, 1H, CH), 3.31 (s, 3H, OCH_3), 3.06 (t, *J* = 9.6 Hz, 1H, CH), 2.19 (s, 3H, CH_3); ^{13}C NMR (150 MHz, $CDCl_3$) δ: 172.8, 146.0, 141.4, 140.3, 129.3, 129.2, 128.8, 128.4, 127.9, 127.5, 127.1, 122.3, 122.1, 114.5, 114.4, 58.8, 54.0, 53.4, 51.8; IR(KBr) υ: 3409, 2374, 2024, 1719, 1637, 1504, 1428, 1352, 1294, 1193, 1085, 990, 812, 769cm^{-1}; HRMS (ESI) Calcd for $C_{24}H_{23}ClN_2NaO_2$ ($[M+Na]^+$): 429.1345. Found: 429.1342.

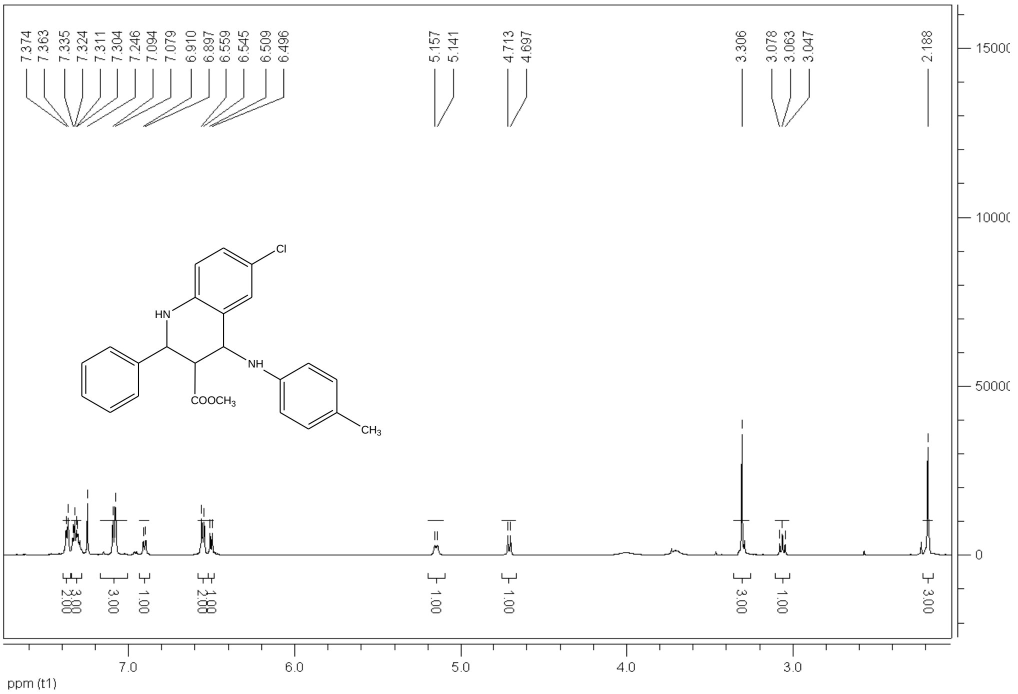

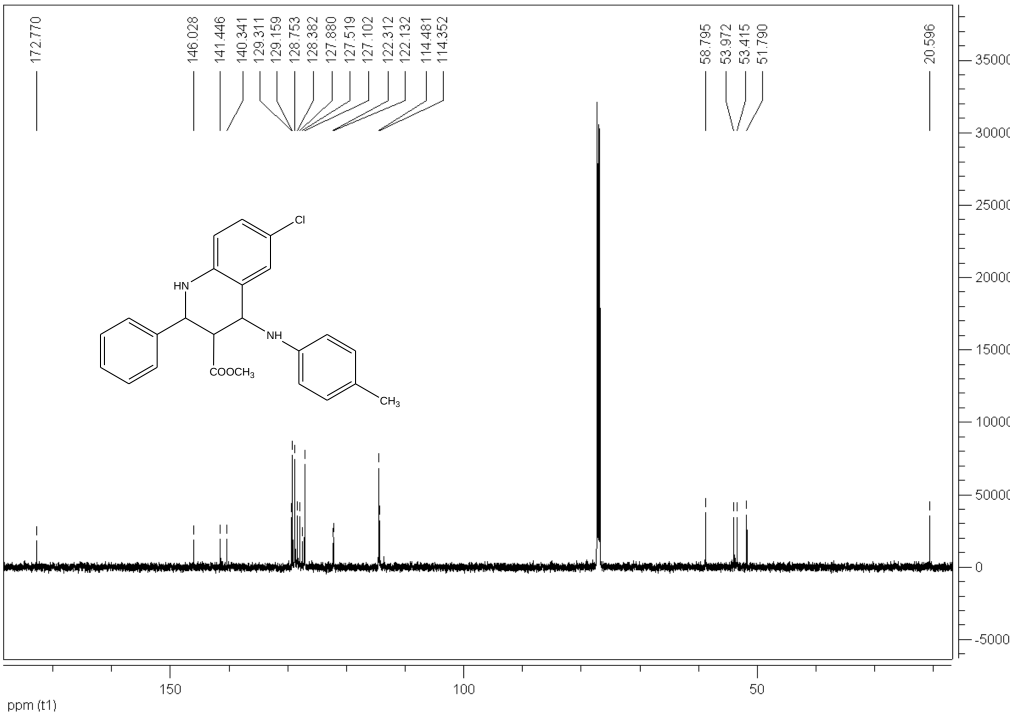

Methyl 6-bromo-2-(4-chlorophenyl)-4-(4-methoxyphenylamino)-1,2,3,4-tetrahydroquinoline-3-carboxylate (2d):

White solid, 57%, mp 170–173℃; ^{1}H NMR (600 MHz, $CDCl_3$) δ: 7.33–7.30 (m, 4H, ArH), 7.22 (d, *J* = 9.0 Hz, 2H, ArH), 6.86 (s, 1H, ArH), 6.70 (d, *J* = 6.0 Hz, 1H, ArH), 6.53 (t, *J* = 8.4 Hz, 3H, ArH), 5.15 (d, *J* = 9.0 Hz, 1H, CH), 4.63 (d, *J* = 9.0 Hz, 1H, CH), 3.65 (s, 3H, OCH_3), 3.31 (s, 3H, OCH_3), 3.02 (t, *J* = 9.6 Hz, 1H, CH); ^{13}C NMR (150 MHz, $CDCl_3$) δ: 172.6, 152.8, 146.2, 138.7, 137.6, 134.1, 132.1, 128.9, 128.6, 123.4, 115.7, 115.1, 112.5, 109.7, 58.6, 55.7, 54.2, 53.5, 51.9; IR(KBr) υ: 3386, 2947, 2833, 2374, 2033, 1722, 1647, 1594, 1498, 1348, 1289, 1196, 1164, 1090, 1042, 955, 875, 805cm^{-1}; HRMS (ESI) Calcd for $C_{24}H_{22}BrClN_2NaO_2$ ($[M+Na]^+$): 523.0399. Found: 523.0393.

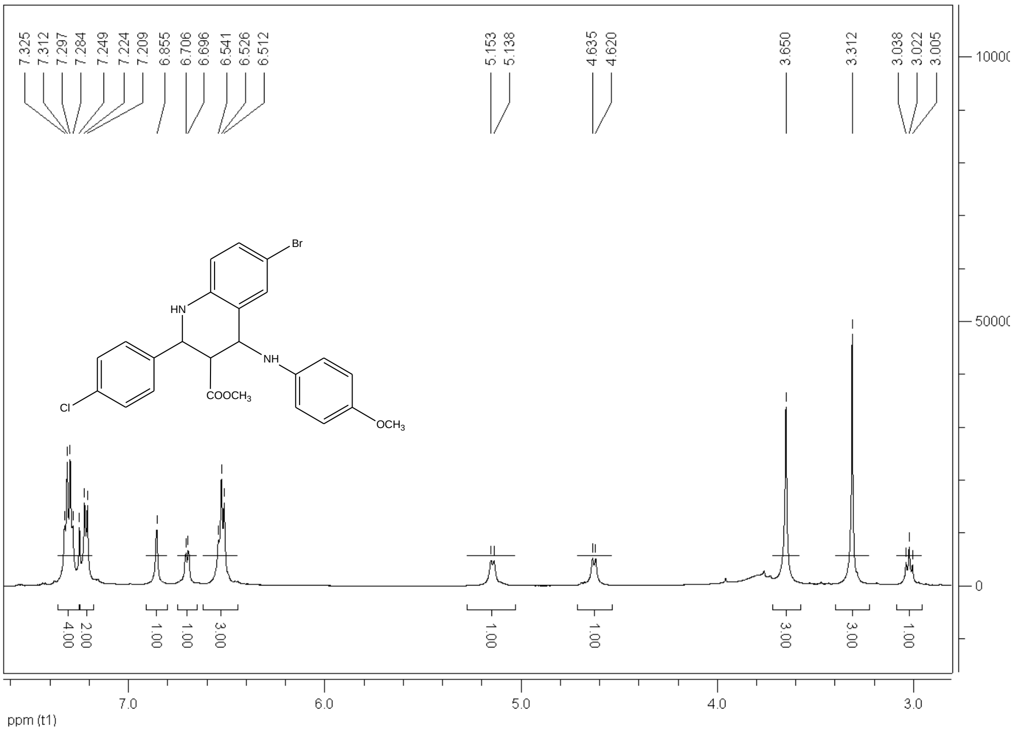

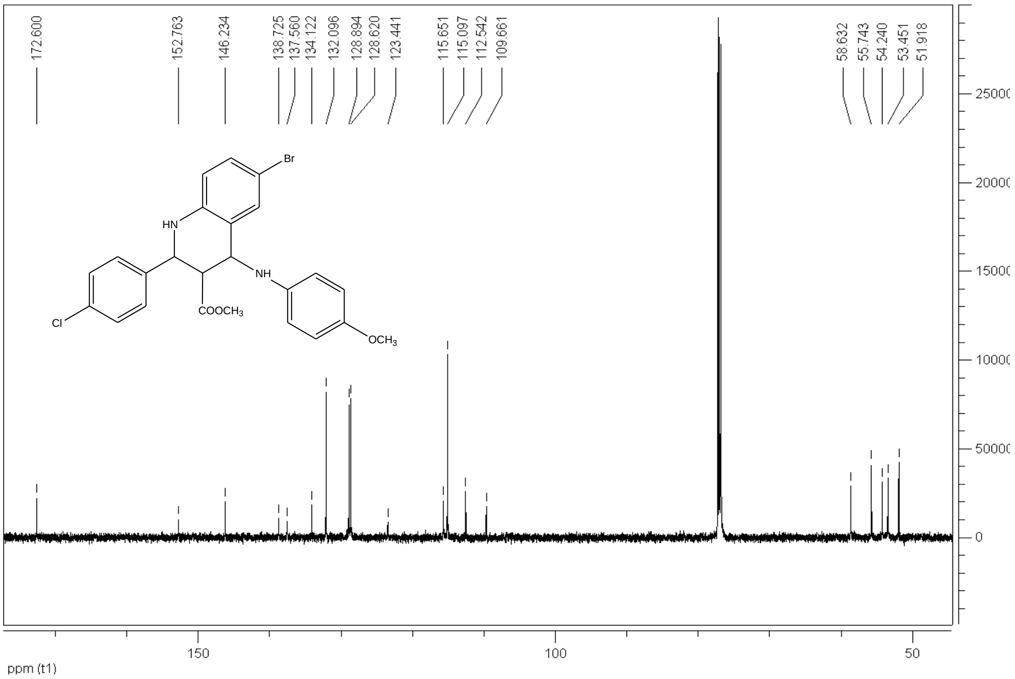

Methyl 2-(4-chlorophenyl)-7-methyl-4-(4-methylphenylamino)-1,2,3,4-tetrahydroquinoline-3-carboxylate (2e):

White solid, 55%, mp 172–175℃; ^{1}H NMR (600 MHz, $CDCl_3$) δ: 7.31 (t, *J* = 8.4 Hz, 4H, ArH), 7.20 (d, *J* = 7.2 Hz, 1H, ArH), 6.96 (d, *J* = 7.2 Hz, 2H, ArH), 6.56 (d, *J* = 7.2 Hz, 2H, ArH), 6.51 (d, *J* = 7.8 Hz, 1H, ArH), 6.38 (s, 1H, ArH), 5.14 (d, *J* = 9.6 Hz, 1H, CH), 4.69 (d, *J* = 9.6 Hz, 1H, CH), 3.32 (s, 3H, OCH_3), 2.99 (t, *J* = 9.6 Hz, 1H, CH), 2.23 (d, *J* = 4.8 Hz, 6H, CH_3); ^{13}C NMR (150 MHz, $CDCl_3$) δ: 172.7, 145.2, 143.4, 139.0, 138.4, 134.1, 129.9, 128.9, 128.6, 127.6, 127.1, 120.4, 119.4, 114.6, 113.5, 58.4, 54.1, 53.8, 51.8, 21.1, 20.4; IR(KBr) υ: 3394, 2947, 2374, 1725, 1619, 1521, 1480, 1433, 1300, 1257, 1163, 1091, 1015, 947, 806cm^{-1}; HRMS (ESI) Calcd for $C_{25}H_{25}ClN_2NaO_2$ ($[M+Na]^+$): 443.1502. Found: 443.1496.

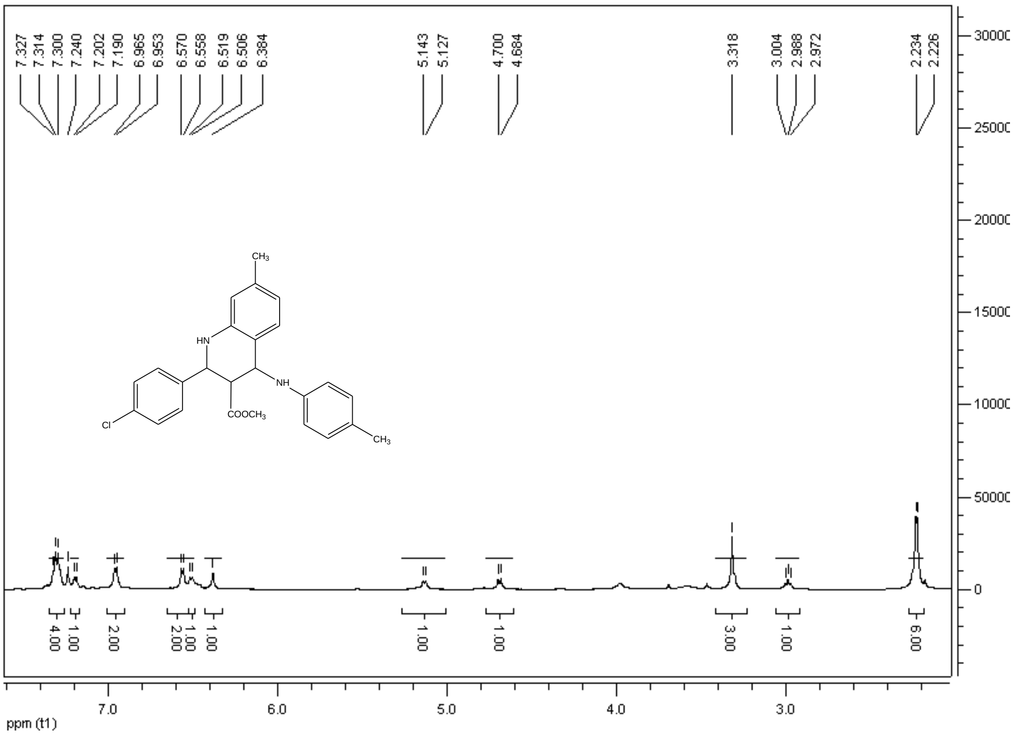

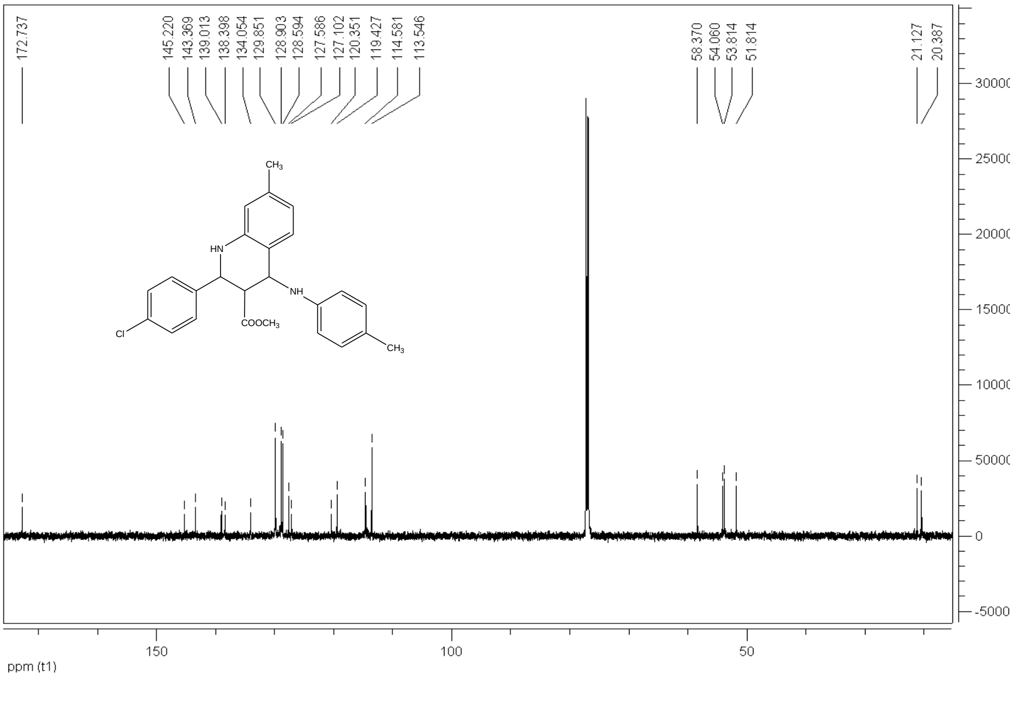

Supplement: File 1 — General experimental methods and characterization of compounds. [file Beilstein_J_Org_Chem-08-1839-s001.pdf]
